# Stock market trading *via* actor-critic reinforcement learning and adaptable data structure

**DOI:** 10.7717/peerj-cs.2690

**Published:** 2025-02-18

**Authors:** Cesar Guevara

**Affiliations:** Quantitative Methods Department, Cunef University, Madrid, Madrid, Spain

**Keywords:** Stock market, Reinforcement learning, Data window structure, Actor-critic, Neural network, Euro, Gold, Crude oil

## Abstract

Currently, the stock market is attractive, and it is challenging to develop an efficient investment model with high accuracy due to changes in the values of the shares for political, economic, and social reasons. This article presents an innovative proposal for a short-term, automatic investment model to reduce capital loss during trading, applying a reinforcement learning (RL) model. On the other hand, we propose an adaptable data window structure to enhance the learning and accuracy of investment agents in three foreign exchange markets: crude oil, gold, and the Euro. In addition, the RL model employs an actor-critic neural network with rectified linear unit (ReLU) neurons to generate specialized investment agents, enabling more efficient trading, minimizing investment losses across different time periods, and reducing the model’s learning time. The proposed RL model obtained a reduction average loss of 0.03% in Euro, 0.25% in gold, and 0.13% in crude oil in the test phase with varying initial conditions.

## Introduction

Stock market trading has been a fruitful area of research for exploring, analyzing, and providing strategies aimed at reaching economic profits and minimizing risks when investing in a market. In finance, a primary objective is to obtain higher profits by allocating resources where they are most effective (Agarwal, Kumar & Goel, 2019). Investment management is based on the perception of the market’s current forecast state and the investors’ experience, making it potentially inefficient and prone to losses caused by the investors’ decision-making (Bustos & Pomares-Quimbaya, 2020).

In recent years, a variety of computational solutions have been proposed to improve investment processes by applying machine learning (ML) methods (Khan et al., 2020). Trading analysis is a process where decisions are made immediately, taking into account market changes, which are highly variable and complex. Such solutions have several advantages over human traders, including their faster execution and not being affected by emotional factors (Charpentier, Élie & Remlinger, 2021). The application of reinforcement learning (RL) to investment modeling is motivated by its ability to address complex and dynamic problems, such as those found in financial markets, where decisions must adapt rapidly to constant fluctuations. As demonstrated in prior studies (Rouf et al., 2021; Kumbure et al., 2022), RL surpasses traditional machine learning approaches by enabling agents to learn directly from interactions with their environment, thereby optimizing investment policies to maximize profits while minimizing losses in real-time. Compared to machine learning approaches, RL allows agents to learn directly from environmental interactions, optimizing investment policies to maximize profits and minimize losses. One of the most helpful ML approaches in financial markets has been RL, which determines the actions that must be carried out to maximize profits from investments (Aboussalah, Xu & Lee, 2021; Lussange et al., 2020). RL methods are frequently grouped into three types: critic-only (Alibekov, Kubalík & Babuška, 2018), actor-only (Wang et al., 2019), and actor-critic (Soleymani & Paquet, 2021) methods.

In the critic-only method, we have many relevant studies such as Li et al. (2019) where researched Temporal difference (TD)-learning in computer science applications. Also, the work published by Jeong & Kim (2019) proposed a Deep Q-learning for stock trading, trading strategy, and transfer learning. In these cases, the critic-only method was used to solve broad optimization problems. Another recent example of the critic-only method in finance can be found in Carta et al. (2021), which proposes a method for investment analysis where preprocessing is carried out through hundreds of deep neural network models; the authors’ approach trains a reward-based classifier to maximize profits, ultimately integrating all decisions and obtaining efficient results. Furthermore, the study published by Ma et al. (2021) details two models that extract information from stock market transactions where fully connected (FC) and long short-term memory layers are used to identify long-term market trends. Leem & Kim (2020) proposes a model based on deep Q-Networks (DQN) for online learning. The results provide an accumulated yield higher than similar studies with a profit range of 16.6–82.8%, which is 39.1% on average. Another Q-learning application is Chakole et al. (2021), in which two ways of representing discrete states in the market environment are proposed. A Q-learning agent identifies the best trading strategies in a dynamic financial market. A league championship algorithm is proposed in Alimoradi & Husseinzadeh Kashan (2018). It performs extraction of stock trading rules for multiple market conditions by applying RL methods with backward Q-learning. This hybrid of two approaches, Sarsa and Q-learning, is used to improve the extraction of optimal trading rules for twenty companies. The results suggest that profits tend to be better when price uptrends are sharp, compared to a genetic network programming method with rule accumulation.

The actor-only method deterministically maps the state to a specific action by parameterizing the actor function (neural network) and updating the actor parameters following the gradient of the policy’s performance, which is called the policy gradient as shown by Yoo et al. (2021). In the work published by Wu et al. (2020), various stock trading dynamic strategies based on deep RL methods applying the actor-only method are proposed. The study uses time series stock market data, and gated recurrent units are applied to identify relevant financial market data features. In addition, autonomous agents are trained to apply Gated Deep Q-learning and Gated Deterministic Policy Gradient trading strategies. They obtained excellent experimental results, showing that their networks have adapted to financial information with a satisfactory generation of financial investment strategies. In the research proposed by Lele et al. (2020), they use a Reinforcement learning-based applying actor-only method approach to develop a trading agent. The method applied different on-policy reinforcement learning algorithms such as Proximal Policy Optimization, Vanilla Policy Gradient, and Trust Region Policy Optimization on the environment to obtain profits. The article published by Fengqian & Chao (2020) proposed deep reinforcement learning applied actor-only method with a candlestick-decomposing features algorithm to execute the high-frequency transaction strategy in the stock market. The experimental results showed that the algorithm obtained higher robustness and prediction accuracy during the test phase.

The actor-critic methods combine the previous methods: a critic estimates the value function to maximize the rewards of the state (*V* value) or the action value (*Q* value), whereas an actor updates the policy distribution in the direction suggested by the critic (such as with policy gradients). An example of this actor-critic method is Troiano, Villa & Loia (2018), which develops a robot applying deep RL with the actor-critic methodology aimed at identifying technical indices and market behavior. The results show that the proposal is viable and that the proposed model obtains efficient precision through simple trading. Another example of the actor-critic is presented by Soleymani & Paquet (2021), which proposes a graph convolutional RL model called deeppocket, which identifies the relationships between variables in the financial market using a graph of the pairwise correlations between assets. The proposed model collects historical information to train an RL actor-critical (AC) agent with two neural networks. The first one is the actor that learns the investment policy; the second one evaluates the best action to optimize the expected investment return. The results obtained are quite efficient over three different investment test periods. In the study presented by Ma, Liu & McAllister (2023), the authors, proposed a Task-Context Mutual actor–critic (TC-MAC) algorithm for portfolio management, which can learn not only the task but also the context of a portfolio. The algorithm uses a mutual actor–critic model to calculate the relationships between local assets’ features and global context embeddings by maximizing mutual information. The obtained results are optimal in terms of multiple metrics and with different test datasets. Another article about the actor-critic published by Schnaubelt (2022) proposed the application of deep reinforcement learning to optimize execution at cryptocurrency exchanges. This proposal uses the learning optimal limit order placement strategies that are highly relevant for both professional asset managers and private investors. The data of this study was 18 months of high-frequency data with more than 300 million trades. The actor-critic model learns cryptocurrency execution strategies from established markets. The results obtained are optimal and adaptable to each market. Nguyen & Luong (2021) analyzed the automatic stock trading problem where applied actor-critic method and a deep deterministic policy gradient technique. The study compares two sets from the US and Vietnam markets, with very different behaviors and trends. Their results suggest the efficiency of deep RL in reducing investment losses. They also comment upon difficulties, such as the variability of the market when the shares do not tend to increase their value. Along the same lines, AbdelKawy, Abdelmoez & Shoukry (2021) detail a deep RL model with multiple autonomous investment agents and interacts with and identifies the behavior of the North American stock market based on a large amount of historical information. The results are very efficient in performance (CPU and GPU) and stock trading. Pham, Luu & Tran (2021) presents the development of a market simulator with multiple agents applying RL with the actor-critic methodology. The agents learn to determine an optimal investment policy and reduce losses by a considerable percentage in a market as dynamic as that of Vietnam.

The article presented by Azhikodan, Bhat & Jadhav (2019) proposes an actor-critic RL model to automate swing trading, applying a deterministic policy gradient. The proposed model aims to maximize the gain in asset value by executing investment actions in the stock market. The study developed a sentiment analysis model using a recurrent convolutional neural network to predict stock trends from financial news across various communication channels. The results obtained by combining the two models showed a 96% precision in prediction and a 1% profit with the RL model.

Table 1 compares numerous studies utilizing the actor-critic RL model, yielding optimal results and profits exceeding 4%. Additionally, three of these studies were implemented in real-time stock markets, while four were laboratory simulations using historical market data. These works represent noteworthy contributions that have enhanced investment and artificial intelligence through the application of various mathematical theorems and techniques.

**Table 1:** Qualitative and quantitative analysis of actor-critic RL models using real-time stock markets test (RTSM), stock markets simulation test (SMST), and application of other methods (AOM) in the investment area.

Articles	Result	RTSM	SMST	AOM
Troiano, Villa & Loia (2018)	Precision > 98%		✓	✓
Soleymani & Paquet (2021)	Profit = 4.47%	✓		✓
Ma, Liu & McAllister (2023)	Profit = 47.72%	✓		✓
Schnaubelt (2022)	shortfalls < 37.71%	✓		✓
Nguyen & Luong (2021)	Profit = 8.15%		✓	✓
AbdelKawy, Abdelmoez & Shoukry (2021)	Profit = 20.7%		✓	✓
Pham, Luu & Tran (2021)	Profit <= 30%		✓	✓
Azhikodan, Bhat & Jadhav (2019)	Profit <= 1%		✓	✓

The study published by Moradi, Weng & Lai (2022) applied an actor-critic reinforcement learning model and stochastic game theory to monitor and maintain the security of electrical network systems. This study identifies the critical sections of the electrical network and develops an efficient strategy to prevent cyberattacks. The RL model proposed uses a deep Q-learning-based stochastic zero-sum Nash strategy solution to reduce the timings of cascading failures in the reward function. The results obtained by the proposed RL model were optimal, with a reduction in cyberattacks of 13.41%.

The article proposed by Razavi et al. (2022) describes a solution for solving the optimal regulation problem for a discrete-time linear time-invariant system using the actor-critic RL model. The RL model, known as the linear quadratic regulation (LQR) problem, guarantees the convergence rate of the state for a system with known dynamics, where the associated Riccati equation is derived. This solution employs the policy iteration (PI) method to solve the LQR problem with a guaranteed convergence rate. The results obtained during the simulations were optimal, with a reduced time guaranteeing a convergence rate of approximately 0.02 s.

Yun et al. (2023) developed a quantum multiagent reinforcement learning (QMARL) algorithm for centralized training and decentralized execution, utilizing an internet connection to control and manage autonomous robots in a smart factory. The proposed algorithm applies the actor-critic RL model, yielding optimal average precision results: 88.4% in phase 1, 72.4% in phase 2, and 97.1% in phases 3 and 4.

The article by Kasaura et al. (2023) presents a comparative analysis between actor-critic RL algorithms, where each action taken by the learning model must comply with specific constraints based on the real-world environment. The study evaluates different variants of RL algorithms for robot control with constraints. The results focus on three main conclusions: First, training the critic RL model with pre-projected actions serves as an optimal baseline for improving performance, especially when penalty terms are considered. Second, using optimization layers and Neural Frank-Wolfe Policy Optimization comes with significant runtime overheads. Finally, mapping techniques are valuable alternatives to optimization layers in the RL model.

Table 2 compares four studies in various scientific fields that applied the actor-critic RL model, including electrical engineering, cybersecurity, and robotics. The results were optimal, showing high precision, reduced processing time, and a high reward rate. These works contribute to different knowledge areas, enhancing the automation of various tasks and processes with excellent results. For this reason, we have selected the actor-critic RL model as the optimal option for our study.

**Table 2:** Qualitative and quantitative analysis of actor-critic RL models using real-time stock markets test (RTSM), stock markets simulation test (SMST), and application of other methods (AOM) in different science areas.

Articles	Results	RTSM	SMST	AOM
Moradi, Weng & Lai (2022)	Ciberattacks: ¡13.41%		✓	✓
Razavi et al. (2022)	$\alpha = 0.80$ and $s = 0.02$		✓	✓
Yun et al. (2023)	Presicion: 85.97%	✓		✓
Kasaura et al. (2023)	Average $reward = 19.70$		✓	✓

### Research motivation

The complexities of stock market investment have prompted the widespread application of machine learning techniques to enhance profitability and mitigate global capital loss across financial institutions. Traditional investment models predominantly rely on technical or fundamental analysis, employing predefined rules that often lack the flexibility to adapt to dynamic market conditions. In contrast, the proposed RL model utilizes an actor-critic architecture, enabling it to learn directly from interactions with the market environment. This approach facilitates continuous adaptation and decision-making informed by historical patterns and real-time market behavior. The contemporary investment landscape is characterized by significant volatility, frequent disruptions driven by political and economic factors, and heightened competition in global markets. Addressing these challenges necessitates the development of models capable of dynamic adaptation and rapid, accurate decision-making, such as the one proposed in this study. For this reason, we have developed an intelligent model to reduce risk during market trades involving gold, the Euro, oil, and the Dow Jones.

This article proposes a model applying actor-critic RL with an efficient neural network topology. Our proposal determines optimal investment actions in the stock market to maximize profits when the price market is up and avoid capital losses when the market is down. Having analyzed the information in stock time series from different markets, we propose a new temporal data window scheme that identifies the optimal size of the training sale in the actor-critic model. This scheme improves the performance of each agent during the investment and reduces training and processing time. In this way, the obtained results reduce the number of unnecessary investment policies during learning and the losses in investment capital. The intended contributions of this article are:

1) Creating an adaptable data structure for a specific period of time to enhance the learning and accuracy of investment agents.
2) Generating specialized investment agents for each stock market and time period to minimize capital loss.
3) Applying the multi-agent actor-critic RL method to improve investments in various time periods and reduce the model’s learning time.

The article is structured as follows. “Materials and Methods” presents the materials that describe the stock market databases used in this research. Also, this section presents the methods applied as the structure of the reinforcement learning actor-critic model. “Reinforcement Learning Model Proposed” describes our solution approach and the data sets used, detailing the structure of the neural networks in optimizing policies and rewards. “Experiments and Results” shows the RL model’s experiments and results obtained in various markets. In addition, we compare and discuss the results with relevant works and buy and hold strategy to determine the efficiency of the proposed model. Finally, conclusions and future research lines are presented.

## Materials and Methods

In this section, we will describe the materials, which consist of financial datasets, and the methods employed, specifically neural networks, to develop the proposed reinforcement learning model.

### Materials

The data utilized in this study were sourced from Yahoo Finance (2024) and encompass historical records for the Crude oil, Gold, and Euro markets, covering the period from 2015 to 2021. These datasets were meticulously preprocessed to ensure completeness, consistency, and suitability for simulations in a realistic experimental environment. The information available includes the date (registration date), open (opening value of market shares), high (highest value), low (lowest value), close (value at closing), and adj close (closing price adjusted for splits and distributions of dividends and/or profit) for each day. Table 3 shows an example of the three stocks (USD).

**Table 3:** Example of stock exchange data structure.

Datasets	Date	Open	High	Low	Close	Adj.Close
Crude oil	02/01/15	53.76	55.11	52.023	52.69	52.69
Gold	02/01/15	1,184.00	1,194.50	1,169.50	1,186.00	1,186.00
Euro	06/01/15	1.19	1.19	1.19	1.19	1.19

The crude oil, gold, and Euro markets were selected for their distinct characteristics and their critical roles in global financial systems. Crude oil represents a highly volatile commodity market, frequently shaped by geopolitical events. Gold, in contrast, serves as a traditional safe-haven asset, characterized by relatively stable price trends. The Euro exemplifies the dynamics of a major currency market, reflecting regional economic policies and global financial interactions. Together, these diverse attributes create a comprehensive and challenging testing environment for assessing the adaptability and effectiveness of the proposed reinforcement learning model.

#### Data preprocessing and visualization data

**Figure 1:**
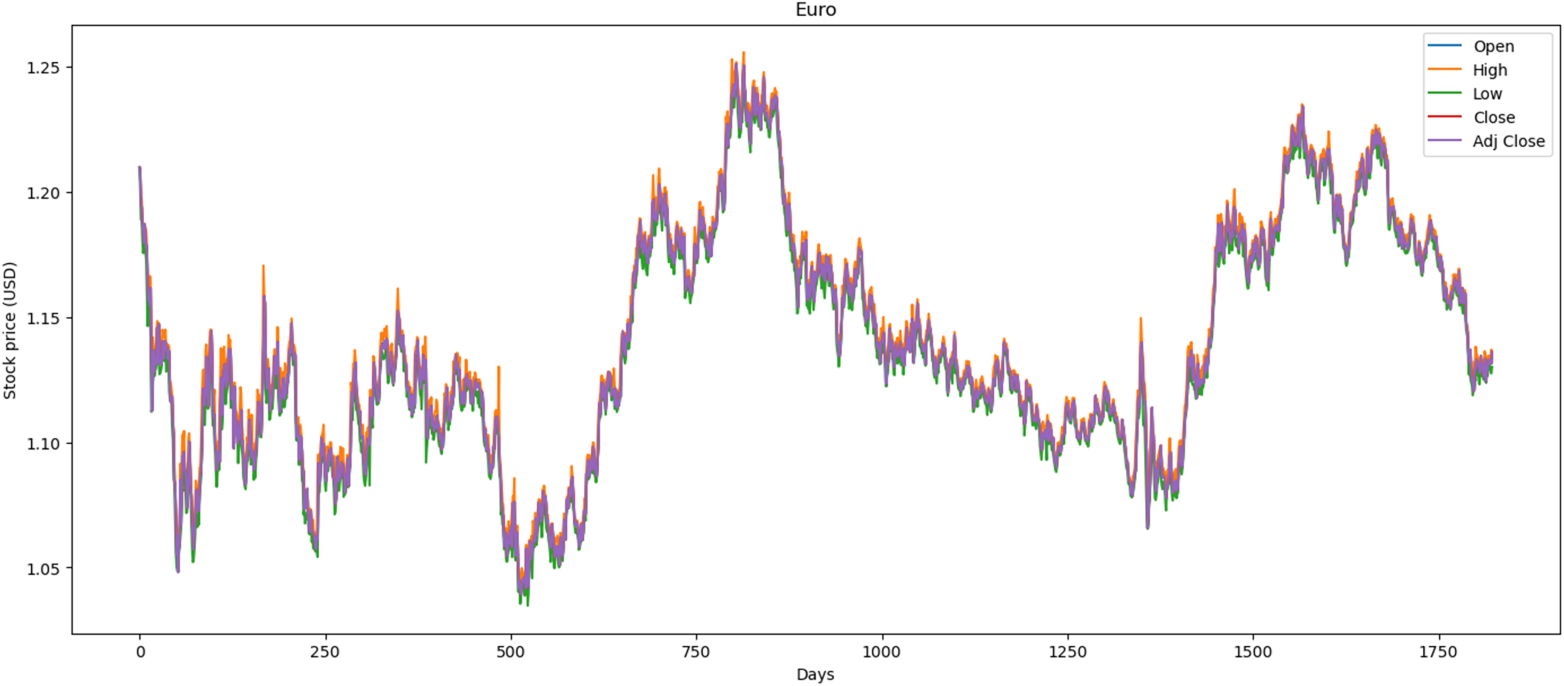
Behavior of Euro stocks market (between 2015 and 2021).

We used the data markets between 01/01/2015 and 31/12/2021, where each market has 1,772 records and in total 5,316 records. In this dataset, we preprocessed the data by correcting numerous errors with negative values, converting the decimal format from point to comma, and changing the date format from month/day/year to day/month/year. Figure 1 illustrates the performance of the Euro market, which comprises 1,772 records. The minimum opening price is $1.04, while the closing price is $1.13. Additionally, it features maximum and average opening and closing prices of $1.25 and $1.14, respectively. Over the past year, there has been a downward trend attributed to political and social changes worldwide.

**Figure 2:**
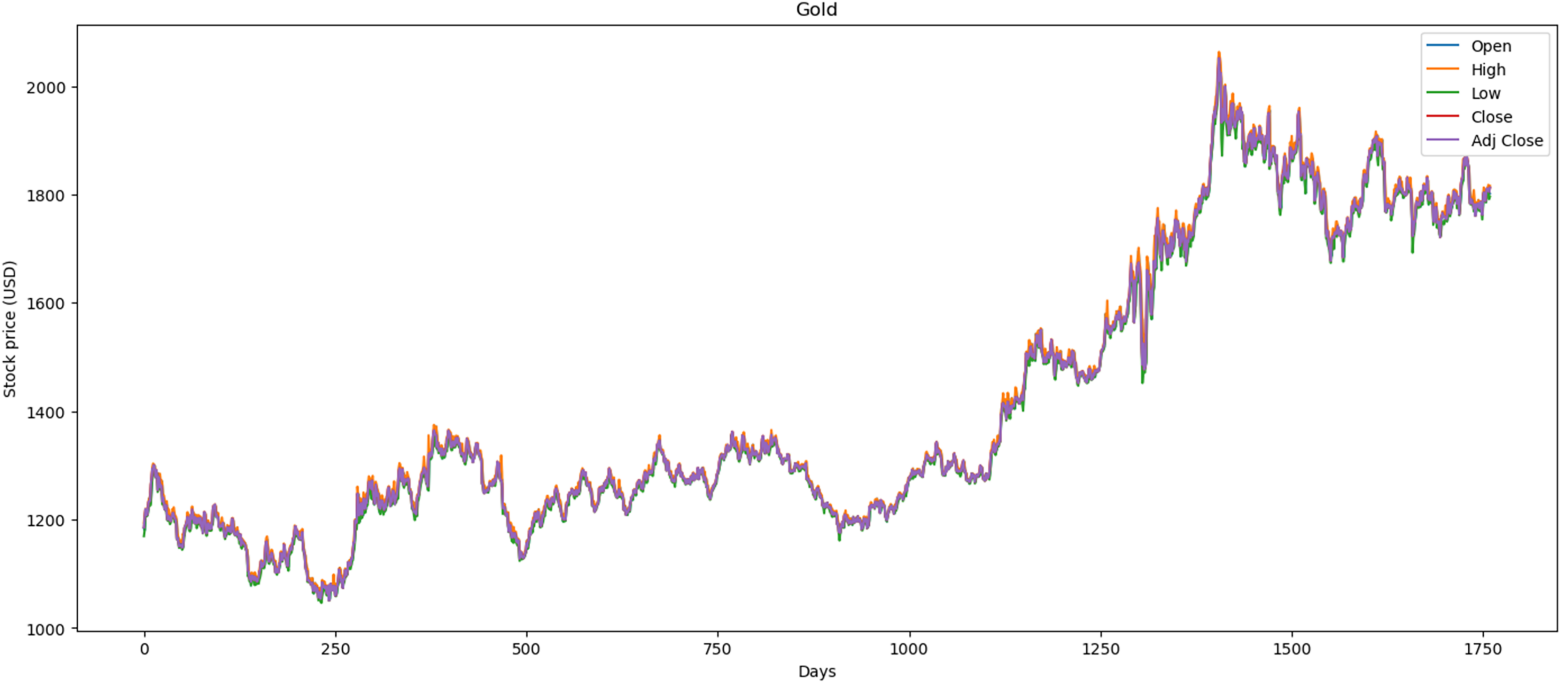
Behavior of gold stocks market (between 2015 and 2021).

Figure 2 illustrates the performance of the gold market, which includes 1,772 records. The minimum opening price is $1,062.00, while the closing price is $1,050.80. Furthermore, it exhibits a maximum opening price of $2,063.00 and a closing price of $2,051.50, with an average opening price of $1,421.36 and a closing price of $1,414.64. Over the past 2 years, price stability has prevailed in the market, largely influenced by gold’s value within the global economy.

**Figure 3:**
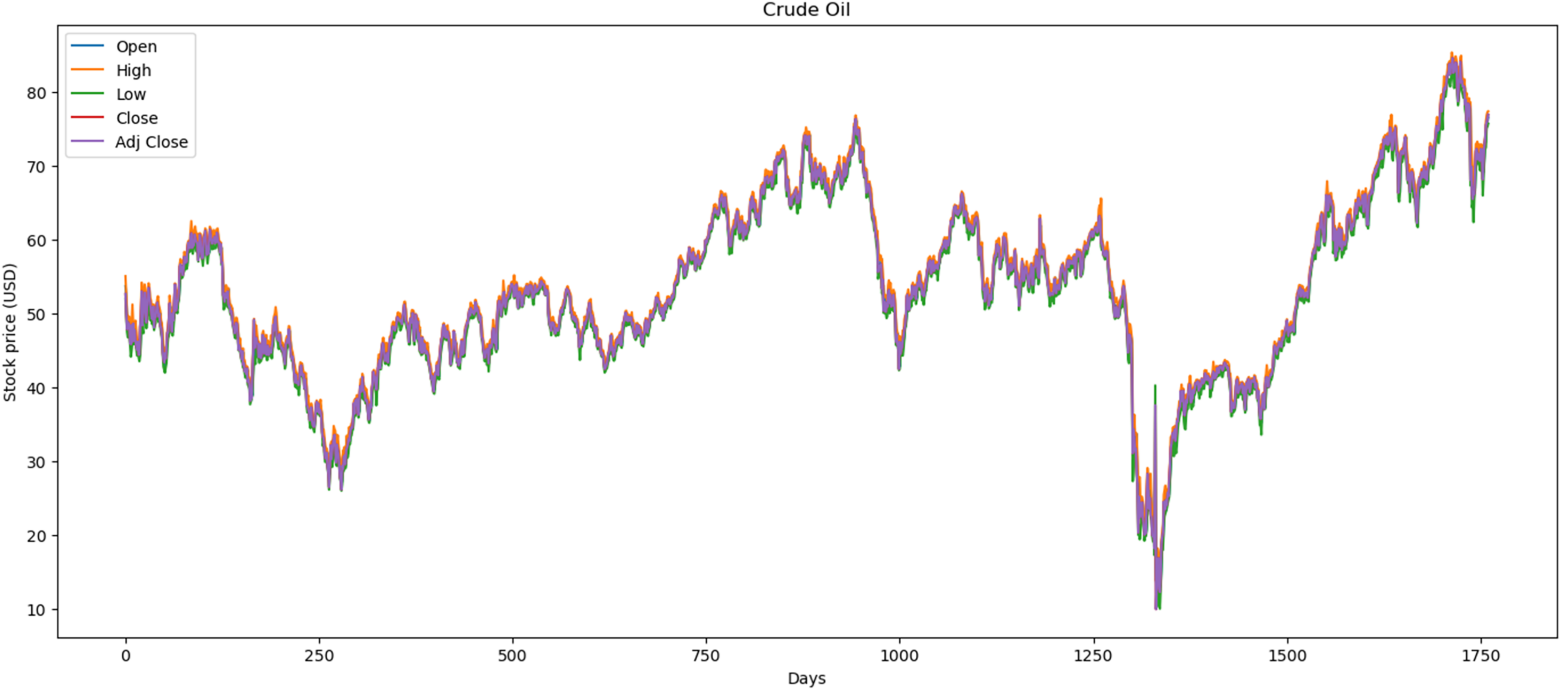
Behavior of crude oil stocks market (between 2015 and 2021).

Figure 3 illustrates the performance of the crude oil market, which encompasses 1,772 records. The minimum opening price is $13.69, while the closing price is $10.01. Additionally, it features a maximum opening price of $85.41 and a closing price of $84.65, with an average opening price of $54.13 and a closing price of $53.26.

### Methods

#### Reinforcement learning actor-critic

In this section, we describe the environment (stock market), the reward, and the actor-critic neural network of the RL model. The model RL aims to support a financial entity *X* in investing in stock market (environment) shares to maximize profits *G* and avoid capital investment losses *C*. The structure of the proposed actor-critic RL model within its environment presents an agent that can execute three investment operations in the market environment: sell, buy, and hold a stock based on its price. Each stock has four values in the market: open value (the first price of the day), close value (the last price of the day), high value (the maximum price of the day), and low value (the minimum price of the day) as shown in “Materials”. The set of possible actions ${A_t}$ is the combination of the number of operations *vs* the number of the values of each stock, where we obtained 27 actions ${A_t}$. With the information on the stock value (three values: close, high, and low), the agent will analyze seven control points of the historical prices of the stocks in the environment. The first control point is stocks owned that the agent currently has (three values). The second control point is the stock value on the day it was bought (three values). The third one is the capital for investment that the agent has (one value). The fourth one is the stock price of the previous day of the current date (three values). The fifth one is the stock value of the last week (workweek), where we can determine the behavior of values in the market in the previous 5 days (three values). The sixth one is the stock value during the last 8 days, through which we can determine the market values in the previous week and a half (three values). Finally, the seventh one is the mean of the stock values in the last 8 days, where we define a threshold of the historical values in the market (three values). With all this information, the agent analyzes 19 observations ${O_t}$ in its RL model. The model incorporated features derived from historical stock values, including opening, closing, high, and low prices, as well as computed indicators such as moving averages and percentage changes in price.

The agent is trained with daily historical values of the shares of interest. At each period $t$, the agent receives observations ${O_t}$ referring to stock values and market rewards ${R_t}$; consequently, it submits an action ${A_t}$ concerning buying or selling stocks. The reward assesses the eventual success of the previous action ${A_{(t - 1)}}$ concerning whether a correct investment has obtained profits.

#### Rewards

To determine the reward ${R_t}$, the AC agent calculates the price rate of change (ROC) indicator (Gupta, Bhattacharjee & Bishnu, 2022), applying the equation

(1) $$Ro{c_t} = {{{O_t} - {O_{t - 1}}} \over {{O_t}}}$$

thus measuring the percentage of change in price between times $t$ and $t - 1$. A positive reward ${R_t} = 1$ is declared when the agent performs an investment action ${A_t} = Buy$ resulting in a profit ( $Ro{c_t}\ \gt\ 0$), which occurs whenever the agent sells the shares when prices begin to fall, avoiding capital losses, or when a stock is held and, as a result, increases its capital. In this study, capital loss refers to the decrease in the total portfolio value resulting from suboptimal investment decisions, such as selling stocks during a price increase or buying during a price decline, as presented by Guo & Ching (2021). This measure is quantified using the negative percentage change in the rate of change (ROC) indicator. However, a negative reward ${R_t} = - 1$ is declared when the agent performs a $Buy$ or $Sell$ action ${A_t}$ resulting in a capital loss ( $Ro{c_t}\ \lt\ 0$). This occurs whenever the agent sells, and the prices rise or buy when prices fall; it also happens whenever the agent performs a $Hold$ action resulting in a portfolio value decrease. Finally, when the agent preserves the stock value ( $Ro{c_t} \ge 0$), the reward is ${R_t} = 0$.

We applied the shaping rewards (Dong, Tang & Yuan, 2020), where the basic idea is to provide small intermediate rewards to the algorithm, aiding it in converging more quickly. The agent learns incrementally, adjusting its behavior based on the intermediate rewards received in subtasks, which facilitates global learning. The shaping rewards implementation in reinforcement learning for stock market investing can help agents learn more effectively by clearly indicating which actions are considered positive for investment success.

#### Actor-critic networks

**Figure 4:**
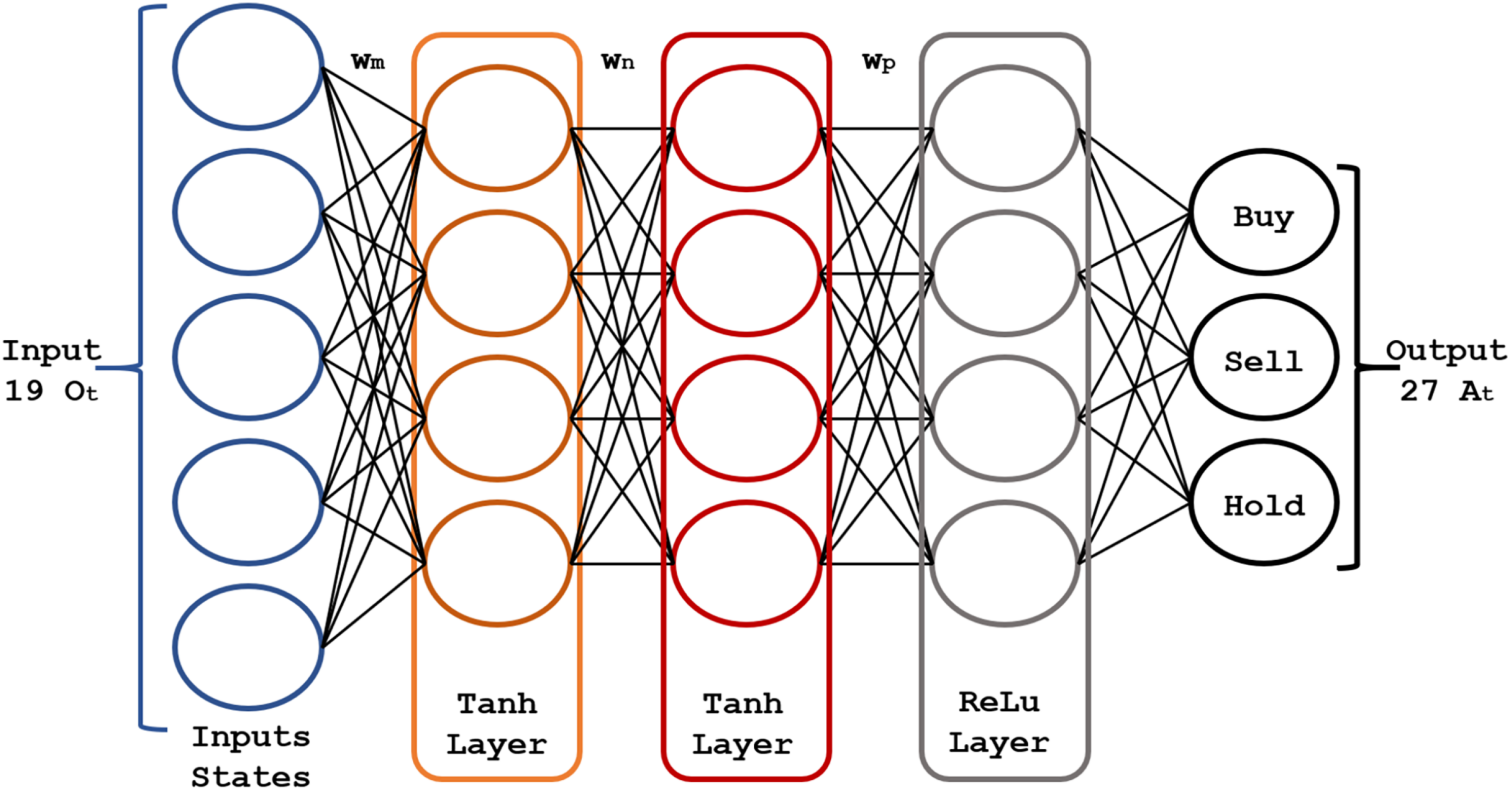
Structure of the actor neural network.

**Figure 5:**
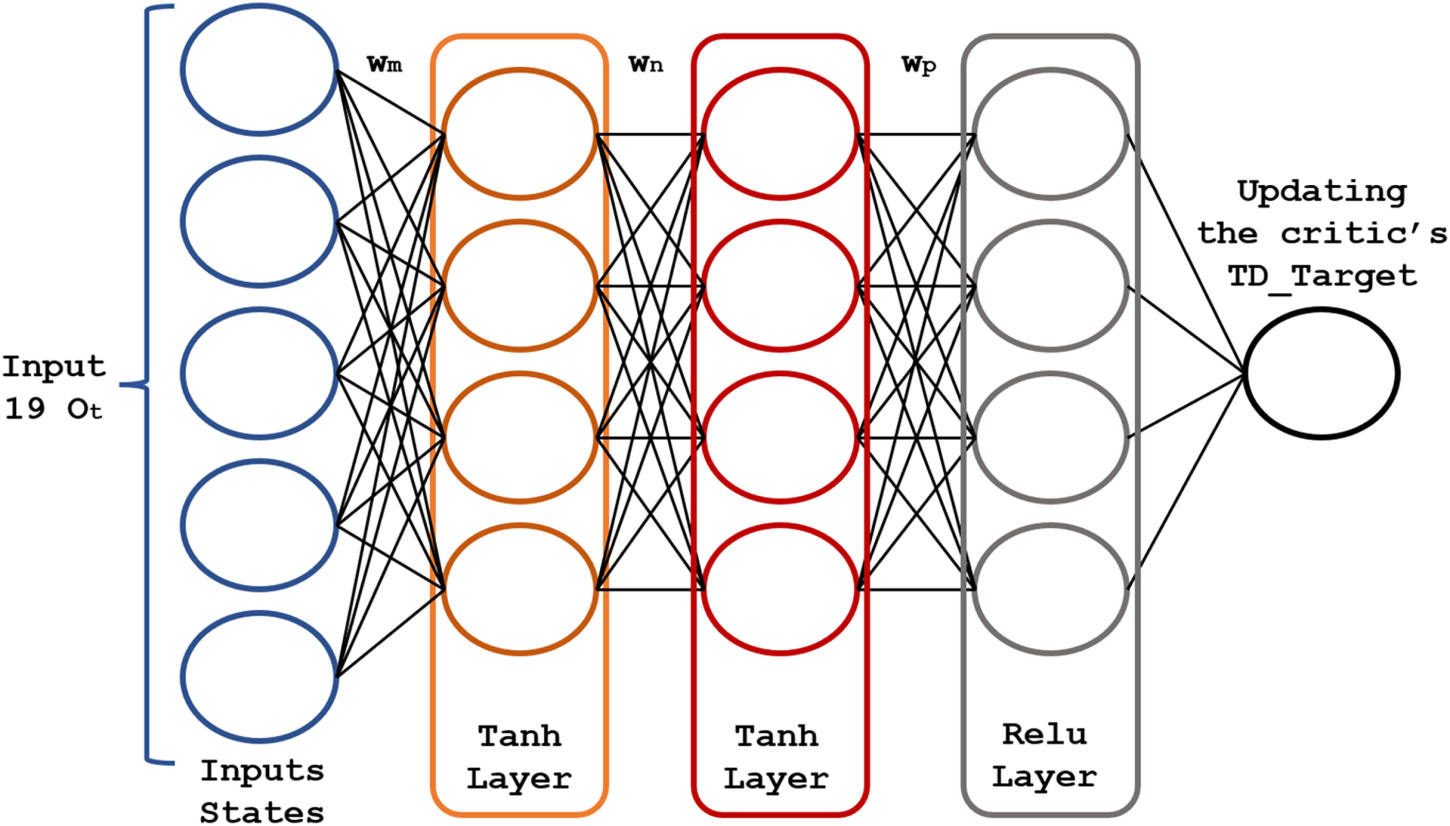
Structure of the critic neural network.

The core of the RL model is a pair of fully connected (FC) neural networks represented in Figs. 4 and 5.

The actor-network chooses an action ${A_t}$ at each time step $t$; in turn, the critic network assesses the decision quality based on the input ${O_t}$. As this network learns which states are better or worse, the actor-network uses this information to teach the agent to look for good states and avoid bad ones. The actor-network learns and updates the weights ${w_n}$, ${w_m}$ and ${w_p}$ of the neural network based on the probabilities $\pi ({A_t}|{O_t};\theta )$, as shown by Soleymani & Paquet (2021).

The actor-network uses 19 inputs (corresponding to the ${O_t}$ 19 states mentioned above) and three hidden layers for the resulting actions ${A_t}$. For the hidden layers, we found the best parametric choices for the activation functions and the number of neurons in each layer using a genetic algorithm. The actor-network maps each state ${O_t}$ generates an output that will be the optimal action ${A_t}$ to invest in the market. Figure 4 shows the scheme of the actor neural network.

The critic network maps each state ${O_t}$ (19 inputs ${O_t}$) to its corresponding action ${A_t}$ value, assessing its quality. This critic network generates the value $T{D_{Target}} = {R_t} + \gamma + V({O_t}^\prime )$ at each learning step, where $T{D_{Target}}$ is the predicted value of all future rewards given the current state ${O_t}$ and $V({O_t}^\prime )$ represents the critical-network assessing the next state value ${O_t}^\prime$. The advantage function is $T{D_{Error}} = T{D_{Target}} - V({O_t})$, which can be interpreted as a prediction error of the AC agent (Singh et al., 2022).

The advantage function describes whether an ${O_t}$ state is better or worse than expected. If an action ${A_t}$ in a state ${O_t}$ is better than expected (advantage greater than 0), the actor-network is encouraged to keep on performing similar actions ${A_t}$. However, if it is worse than expected (advantage less than 0), the actor-network is encouraged to adopt other actions. If an action performs exactly as expected (advantage equals 0), the actor-network learns nothing from that action ${A_t}$.

The critic network (Fig. 5) consists of three FC layers with $Tanh$ and $ReLu$ activation functions. Its output is $T{D_{Target}}$, optimized using the mean squared error loss function. Its weights ${w_m},{w_n}$, and ${w_p}$ are adjusted (updated) to the new value $T{D_{Target}}$, after each time step $t$. Rectified linear unit (ReLU) neurons were chosen due to their computational efficiency and their effectiveness in mitigating the vanishing gradient problem. This property is particularly critical for optimizing deep neural networks in reinforcement learning applications, where frequent and rapid updates are essential for model performance. To address overfitting, regularization techniques, dropout applied to hidden layers, and temporal cross-validation were employed to enhance the model’s ability to generalize to unseen markets.

The actor-critic method integrates two complementary components: the actor, which determines the optimal action to take in a given state, and the critic, which assesses the quality of these actions by estimating a value function. These components operate in tandem to refine the decision-making policy through the policy gradient and the advantage function, ultimately aiming to maximize long-term cumulative rewards. An actor-critic agent during training estimates probabilities of taking each action ${A_t}$ in the action space and randomly selects actions based on the probability distribution. The agent interacts with the environment for multiple steps using the current policy before updating the actor and critic properties. An actor-critic agent maintains two function approximators to estimate the policy and value function; first, actor $\pi ({A_t}|{O_t};\theta )$ with parameters $\theta$, outputs the conditional probability of taking each action ${A_t}$ when in state ${O_t}$ as a discrete action space where the sum of these probabilities across all actions is 1. Second, critic $V(O,\varphi )$, with parameters $\varphi$, takes observation ${O_t}$ and returns the corresponding expectation of the discounted long-term reward. However, during training, the agent adjusts the parameter values in $\theta$. At the end of the training, the parameters remain tuned, and the trained actor function approximator is stored in $\pi ({A_t}|{O_t};\theta )$.

Actor-critic agents use the following training process by applying nine configuration steps. In the first step, we initialize the actor $\pi ({A_t}|{O_t};\theta )$ with random parameter values $\theta$. In the second step, we initialize the critic $V(O,\varphi )$ with random parameter values $\varphi$. The third step generates *N* episodes by following the current policy, defined as

(2) $${O_{to}},{A_{to}},{R_{(to + 1)}},...,{O_{(to + N - 1)}},{A_{(to + N - 1)}},{R_{(to + N)}},{O_{(to + N)}}$$

where ${O_t}$ is a state observation, ${A_t}$ is an action taken from that state, ${O_t} + 1$ is the next state, and $Rt + 1$ is the reward received for moving from ${O_t}$ to ${O_t} + 1$. In state ${O_t}$, the agent computes the probability of taking each action in the action space using $\pi ({A_t}|{O_t};\theta )$ and randomly selects action ${A_t}$ based on the probability distribution. The starting-time step of the current set of *N* episodes is $to$, where the beginning of the training episode is $ts = 1$ and the terminal state is ${O_{(to + N)}}$. For each following set of *N* episodes in the same training episode, $to = to + N$.

In the fourth step for each episode step $t = {t_o} + 1,{t_o} + 2,...,{t_o} + N$, the return is computed according to

(3) $${G_t} = \sum\limits_{k = t}^{{t_o} + N} {({\gamma ^{(k - t)}}{R_k})} + b{\gamma ^{(N - t + 1)}}V({O_{({t_o} + N)}};\varphi ),$$

which is the sum of the reward for that step and the discounted future reward. If ${O_{(to + N)}}$ is not a terminal state, the discounted future reward includes the discounted state value function, computed using the critic network *V*. However, $b$ is $0$ if ${O_{({t_o} + N)}}$ is a terminal state and $1$ otherwise. The discount factor is defined as $\gamma$.

The fifth step computes the advantage function ${D_t} = {G_t} - V({O_t},\varphi )$. In the sixth step, it accumulates the gradients for the actor-network by following the policy gradient to maximize the expected discounted reward, as

(4) $$d\theta = \sum\limits_{t = 1}^N \nabla {\theta _\mu }\ln \pi ({A_t}|{O_t};\theta ){D_t}$$

In the seventh step, it accumulates the gradients for the critic network by minimizing the mean squared error loss between the estimated value function $V({O_t},\varphi )$ and the computed target return $Gt$ across all *N* episodes, as

(5) $$d\varphi = \sum\limits_{t = 1}^N \nabla \varphi {({G_t} - V({O_t};\varphi ))^2}.$$

In the eighth step, it updates the actor parameters by applying the gradients, defined as $\theta = \theta + \alpha d\theta$, where $\alpha$ is the learning rate of the actor. The ninth step updates the critic parameters by applying the gradients as $\varphi = \varphi + \beta d\varphi$, where $\beta$ is the critical learning rate. Finally, this process is repeated from step 3 to step 9 for each training episode until training is complete, as presented by Mnih et al. (2016).

## Reinforcement learning model proposed

This section describes the relevant contributions of the investment area applying RL. Actor-only, critic-only, and actor-critic methods were evaluated for this study. The actor-critic approach was selected due to its capacity to balance action evaluation and state assessment, thereby optimizing both the policy and the expected rewards. To generate the RL proposed model, it was necessary to identify the appropriate data sets for achieving optimal performance in training and testing. With this goal in mind, we generated agents depending on the specific time series; that is, we trained each agent for specific periods named adaptable data structure (semester, trimester, 4-month period, *etc*.).

**Figure 6:**
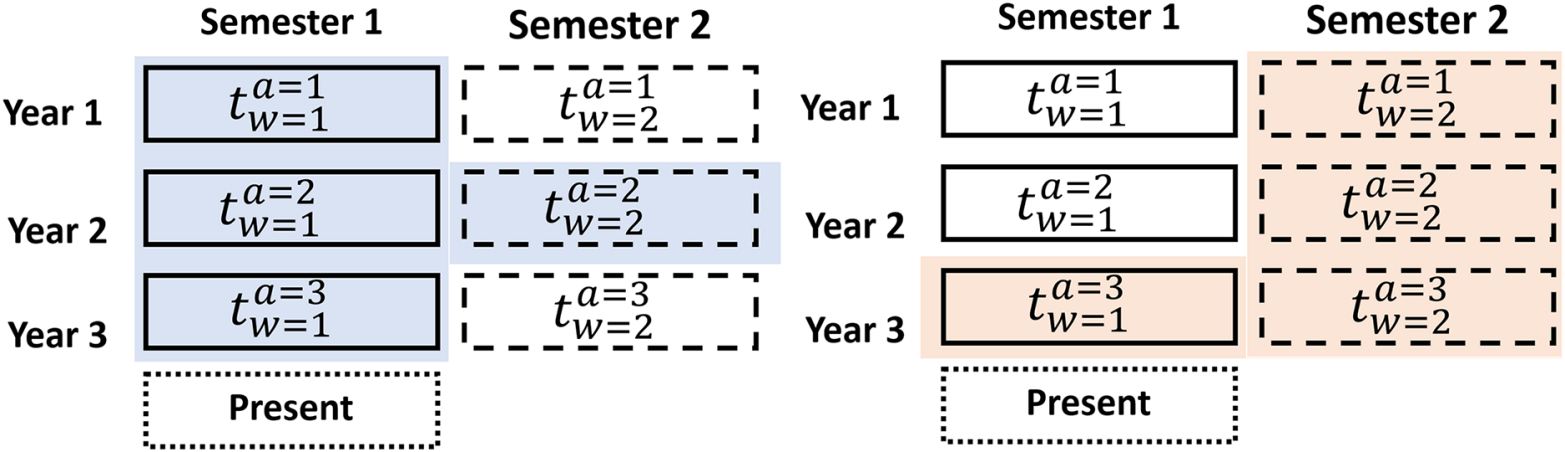
Adaptable data structure proposed for a specific period.

The data set *D* has historical information on a product or sector of the stock market. Each data set contains several groups of data $a$ with a defined number $n$ of years in the historical data, where the data set is $D = \{ {a_1},{a_2},...,{a_n}\}$. Each data year ${a_n}$ can be divided into different periods, named dynamic data windows $tw$, that divide the year into 2, 3, 4, 6, or 12 parts, where ${a_n} = \{ t{w_1},t{w_2},...,t{w_{mt}}\}$ and $mt$ is the number of parts. As shown in Fig. 6, we train an actor-critic agent through the adaptable data structure $tw$ at a specific period based on Guevara, Santos & López (2017) which is the first contribution of the research.

**Figure 7:**
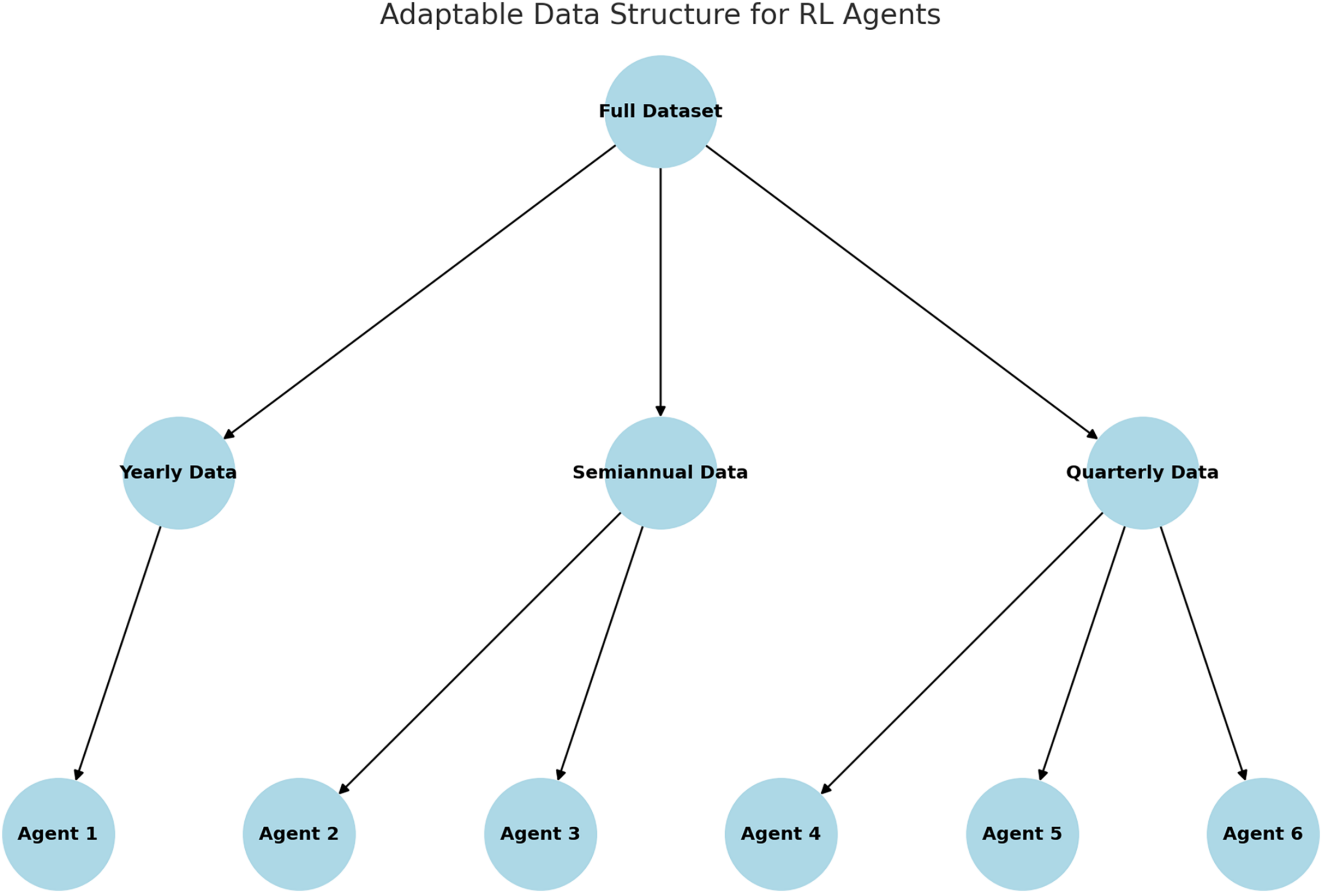
Adaptable data structure proposed.

Figure 7 illustrates the adaptive data window structure utilized in the RL model. The dataset is partitioned into annual, semi-annual, and quarterly subsets, with specialized agents assigned to each time window. This approach enhances the agents’ ability to capture and learn patterns specific to individual periods, thereby improving their overall performance.

**Algorithm 1:** Pseudocode for adaptive data structure in RL agents.

1: **Input:** Full dataset *D*, window configurations $windows = \{ {\mathrm{yearly}},\;{\mathrm{semiannual}},\;{\mathrm{quarterly}}\}$
2: **Output:** Trained agents for specific time periods
3:
4: **Function** TrainRLAgnets (*D*, *windows*):
5: $data\_splits \leftarrow$ SplitDataset(*D*, *windows*)
6: $agents \leftarrow \{ \}$ {Initialize empty dictionary for storing agents}
7: **for each** $window\_type$ **in** *windows*:
8: **for each** *segment* **in** $data\_splits[window\_type]$:
9: $agent \leftarrow$ InitializeRLModel()
10: Train(*agent*, *segment.data*) {Train agent on data segment}
11: $agents[segment] \leftarrow agent$
12: **return** *agents*
13:
14: **Function** SplitDataset(*D*, *windows*):
15: $data\_splits \leftarrow \{ \}$
16: **for each** $window\_type$ **in** *windows*:
17: **if** $window\_type = =$ “yearly”:
18: $data\_splits[window\_type] \leftarrow$ SplitByYear(*D*)
19: **if** $window\_type = =$ "semiannual":
20: $data\_splits[window\_type] \leftarrow$ SplitBySixMonths(*D*)
21: **if** $window\_type = =$ "quarterly":
22: $data\_splits[window\_type] \leftarrow$ SplitByThreeMonths(*D*)
23: **return** $data\_splits$
24:
25: **Main Program**:
26: $D \leftarrow$ LoadMarketData()
27: $windows \leftarrow \{ {\mathrm{yearly}},\;{\mathrm{semiannual}},\;{\mathrm{quarterly}}\}$
28: $trained\_agents \leftarrow$ TrainRLAgents(*D*, *windows*)
29: Print(“Agents trained:", $trained\_agents.keys()$)

The pseudocode 1 outlines the implementation of an adaptive data window structure for training RL agents specialized in specific time periods. The complete dataset *D* is partitioned into dynamic subsets defined by temporal configurations, such as annual, semi-annual, and quarterly, which serve as inputs to the algorithm. For each configuration, the data is further segmented into specific time windows and assigned to independent agents. Each agent is trained exclusively on the data corresponding to its designated time window, enabling it to capture period-specific patterns and enhance its decision-making capabilities. This modular approach effectively addresses changing market dynamics, as specialized agents are better equipped to adapt to the unique characteristics of their respective time segments.

The process begins by loading the historical market dataset and defining the dynamic data window configurations. The dataset is then segmented into subsets according to the selected temporal configuration. For each segment, an RL model is initialized and trained using the actor-critic method with the data corresponding to the specific time period. The trained agents are stored as independent units, ready for deployment in real-world scenarios. This methodology not only improves training accuracy and efficiency but also reduces computational requirements by limiting the data processed by each agent.

As an example, suppose the RL model has been trained with three ( $n = 3$) years of data split into 6-month periods ( ${t_{w = 2}}$). The first agent is trained with ${D_{tw = 1}} = t_{w = 1}^{a = 1},t_{w = 1}^{a = 2},t_{w = 2}^{a = 2},t_{w = 1}^{a = 3}$, as shown in Fig. 6 (blue). The second agent is trained with ${D_{tw = 2}} = t_{w = 2}^{a = 1},t_{w = 2}^{a = 2},t_{w = 2}^{a = 3},t_{w = 1}^{a = 3},t_{w = 2}^{a = 3}$, as shown in Fig. 6 (orange).

With these datasets ${D_{t{w_{mt}}}}$, the agents require only a small amount of current data to enhance their training and performance. Market variability was addressed using an adaptive data structure, enabling the training of specialized agents for specific periods. This approach reduced the model’s sensitivity to abrupt changes in market conditions. The advantage of an adaptable data structure is that it increases accuracy in decision-making because dataset usage allows for the definition of agents with more specific information. The data structure utilizes an additional data window ( $t_{w = 2}^{a = 2}$ and $t_{w = 1}^{a = 3}$) preceding the last historical data period, providing more up-to-date information on current market behavior. Furthermore, with this contribution reduces training time and the utilization of computational resources. The adaptive data window structure was developed by partitioning the historical datasets into specific periods (*e.g*., semi-annual, quarterly). This approach enabled the identification of optimal configurations through iterative testing applying a genetic algorithm, which aimed to maximize accuracy while minimizing training time.

**Figure 8:**
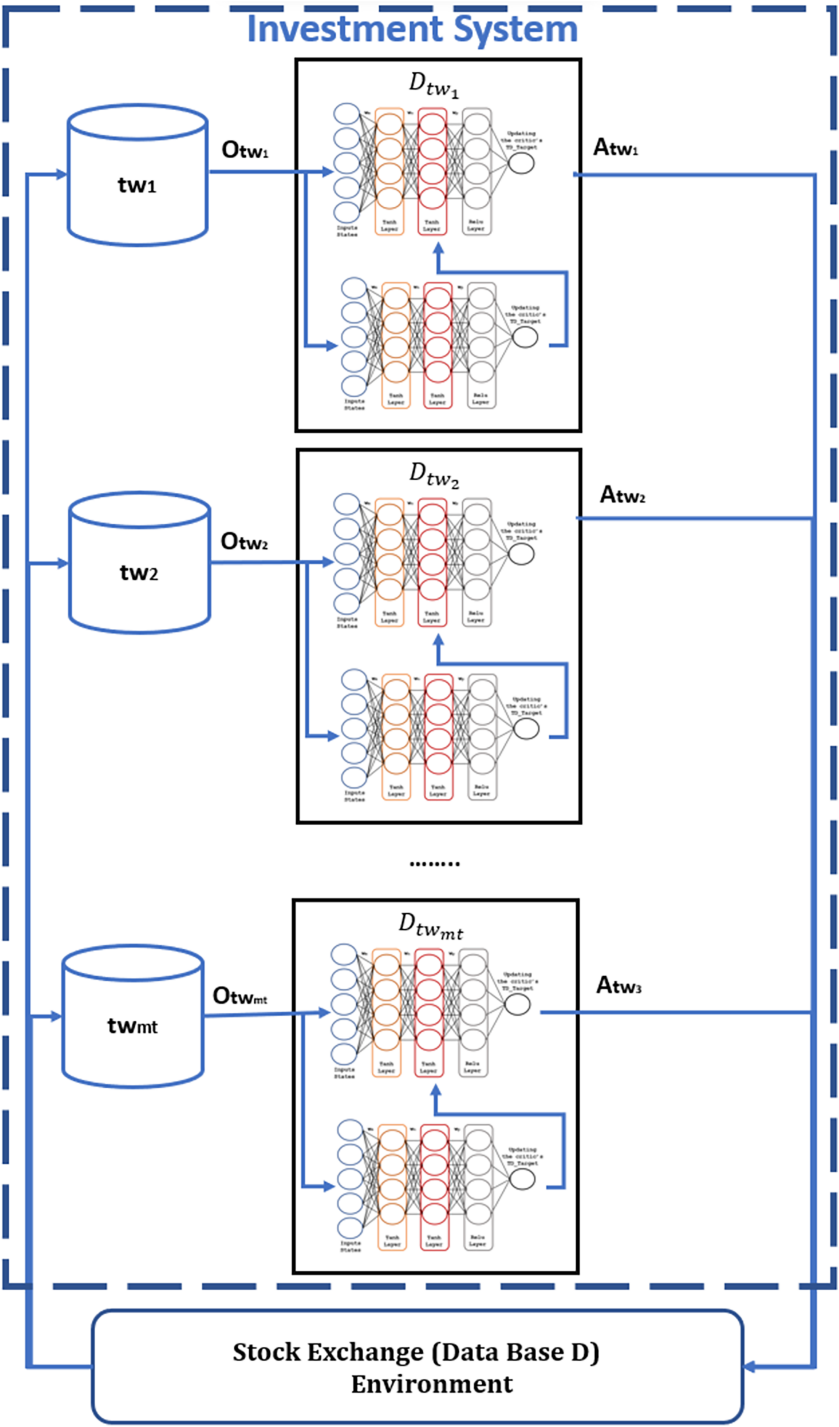
Specialize agents to each period of time for RL model.

Finally, the third proposed contribution allows the definition of multi-agents with more specific information in the actor-critic RL model (Fig. 8), whose investment behavior reduces the loss of capital *C* during trading. These agents work sequentially and only learn within a specific period $tw$, reducing the model’s learning time, based on the adaptable data structure proposed for each stock market.

The adaptive data window structure leverages dynamically divided periods (*e.g*., semi-annual or quarterly) to train specialized agents on distinct market behavior patterns. As illustrated in Fig. 6, earlier periods provide valuable historical context, while more recent periods enable adaptation to current market trends. This approach improves the agents’ capacity to recognize both short- and long-term patterns, thereby enhancing the effectiveness and precision of investment decisions.

## Experiments and results

In this section, we will conduct numerous experiments by applying the actor-critic RL model and the contributions outlined in “Reinforcement Learning Model Proposed”.

### Experiments settings

For these experiments, we will start with an initial capital of $20,000. Additionally, to identify the best version of the proposed model, we will utilize several dynamic data windows, denoted as $tw$. These windows will include annual ( $an$) data sets (one data set), semiannual ( $sm$) data sets (two data sets with 6 months per year each), and quarterly ( $qu$) data sets (three data sets with 4 months per year each). Referring to the scheme presented in Fig. 6, we have obtained the corresponding data sets for each stock as listed in Table 4.

**Table 4:** Dynamic data windows $tw$ in annual $an$, semiannual $sm$, and quarterly $qu$ setups for AC agents.

Window	$tw$	${D_{t{w_{sm = 0}}}}$
Annual	$t{w_{an = 1}}$	${D_{t{w_{an = 1}}}} = \{ tw_{an = 1}^{{a_1}},tw_{an = 1}^{{a_2}},tw_{an = 1}^{{a_3}},tw_{an = 1}^{{a_4}},tw_{an = 1}^5\}$
Semiannual	$t{w_{sm = 1}}$	${D_{t{w_{sm = 1}}}} = \{ tw_{sm = 1}^{{a_1}},tw_{sm = 1}^{{a_2}},tw_{sm = 1}^{{a_3}},tw_{sm = 1}^{{a_4}},tw_{sm = 2}^{{a_4}},tw_{sm = 1}^5\}$
	$t{w_{sm = 2}}$	${D_{t{w_{sm = 2}}}} = \{ tw_{sm = 2}^{{a_1}},tw_{sm = 2}^{{a_2}},tw_{sm = 2}^{{a_3}},tw_{sm = 2}^4,tw_{sm = 1}^5,tw_{sm = 2}^5\}$
Quarterly	$t{w_{qu = 1}}$	${D_{t{w_{qu = 1}}}} = \{ tw_{qu = 1}^{{a_1}},tw_{qu = 1}^{{a_2}},tw_{qu = 1}^{{a_3}},tw_{qu = 1}^{{a_4}},tw_{qu = 3}^{{a_4}},tw_{qu = 1}^5\}$
	$t{w_{qu = 2}}$	${D_{t{w_{qu = 2}}}} = \{ tw_{qu = 2}^{{a_1}},tw_{qu = 2}^{{a_2}},tw_{qu = 2}^{{a_3}},tw_{qu = 2}^4,tw_{qu = 1}^5,tw_{qu = 2}^5\}$
	$t{w_{qu = 3}}$	${D_{t{w_{qu = 3}}}} = \{ tw_{qu = 3}^{{a_1}},tw_{qu = 3}^{{a_2}},tw_{qu = 3}^{{a_3}},tw_{qu = 3}^4,tw_{qu = 2}^5,tw_{qu = 3}^5\}$

To carry out the training of the agents, we needed to configure the structure of their neural networks using the parameters in Table 5. The input and output layers have a number of neurons defined by the observations ${O_t}$, the actions ${A_t}$, and the update of $T{D_{Target}}$ (described in “Actor-critic networks”) for both actor-critic neural networks. The selection of the number of hidden neurons and layers was the optimal configuration after many experiments. The structure of this neural network (Figs. 4 and 5) contains three hidden layers, with 150 neurons in the Tanh layer and one hidden layer with 100 neurons in the ReLu layer. The Tanh and ReLu activation functions were selected based on earlier studies concerning the multilayer neural networks training for RL (Carapuço, Neves & Horta, 2018; Pham, Luu & Tran, 2021).

**Table 5:** Parameter configuration of the actor-critic neural networks and agent.

Parameter	Actor network	Critic network
${X_i}$ Input layer size	19	19
Hidden layer ${L_1}$	150	150
Activation function ${L_1}$	Tanh	Tanh
Hidden layer ${L_2}$	150	150
Activation function ${L_2}$	Tanh	Tanh
Hidden layer ${L_3}$	100	100
Activation function ${L_3}$	ReLu	ReLu
${Y_i}$ Output layer	27	1
AC agent parameters		
Number of steps to look ahead	70	70
Learning rate	0.001	0.001
Entropy loss weight	0.25	0.25
Gradient threshold	1	1
Discount factor	0.91	0.91
Max number of episodes	5,000	5,000
Max steps per episode	4,000	4,000

The model hyperparameters are described in Table 5, such as the parameter Number of steps to look ahead represents the number of steps the agent interacts with the environment before learning from experience, which was finally fixed at $70$. The Entropy loss weight is a value that promotes agent exploration by applying a penalty (between 0 and 1) for being too sure about which action ${A_t}$ to take; this variable facilitates avoiding local optima. Gradients are calculated during training, and an extra gradient component is computed to minimize this loss function. Mnih et al. (2016) suggests a value of $0.25$. The Discount factor is applied to future rewards during training, with a value between 0 and 1. In our experiment, we used $0.91$ in Learning rate to define the learning during training: if close to zero, it leads to a very long training time, whereas a value near one may lead to premature convergence; typical values in the literature range between $0.001$ and $0.002$, and we used $0.001$ (Mnih et al., 2016). The Gradient threshold enables networks to be trained quickly, usually not impacting the learned task accuracy (Pascanu, Mikolov & Bengio, 2013); we used a value of $1$. The Maximum number of episodes is the maximum number of training cycles for the agent, after which training terminates. The “maximum number of steps” used was $\hbox{5,000}$ for the first parameter and $\hbox{4,000}$ for the second parameter, based on AbdelKawy, Abdelmoez & Shoukry (2021).

**Figure 9:**
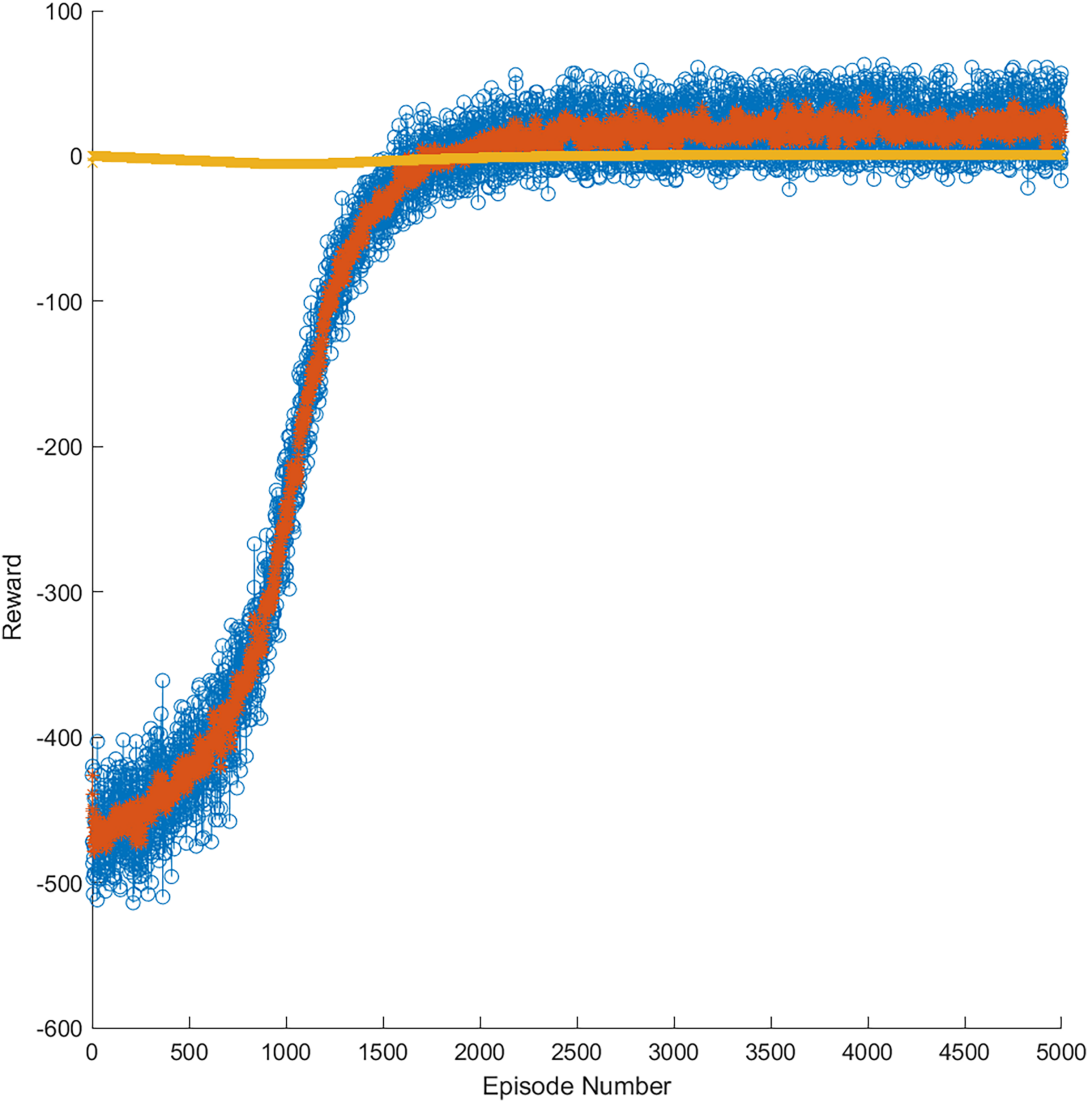
Reward evolution in the gold market in 5,000 episodes.

As mentioned, in the training phase, data windows of annual, semiannual, and quarterly data were used for each market. Figure 9 presents an example of the progress of the learning curve during training until the trained model obtained 5,000 episodes in the gold market. The yellow line represents the optimal threshold limit of learning, whereas the orange line represents the reward per episode during training. In addition, the blue points are the reward episodes during training, having obtained successful results between 1,500 and 5,000 episodes. This training allowed for generating a model more accurate for a price variation in the stock market through RL.

### Evaluation method

To evaluate the performance of an RL actor-critic model applied to neural networks, we used a time series validation approach based on a financial context in which the data exhibited temporal dependence. The historical data of the gold, Euro, oil, and Dow Jones markets were divided into a training dataset containing the first 5 years for each market and a testing dataset with the last 2 years for each market to evaluate the model’s performance. The RL model’s agent was trained to reduce capital loss over time by considering investment actions (sell, buy, and hold). The model observes the accumulated reward at the end of each trading episode. The simulation environment was developed with all financial variables to resemble a realistic trading environment as much as possible. Additionally, a rolling window approach was applied, where the RL model was trained with a dynamic data window to evaluate future data in each iteration.

The model’s performance was validated using a time-series validation approach, where the dataset was divided into training and testing subsets. Furthermore, a rolling window technique was employed to iteratively evaluate the model’s predictions on future data, ensuring robustness and reliability across diverse market scenarios.

### Selection method

Fine-tuning of the RL model was conducted through a hyperparameter search using genetic algorithm optimization. This process aimed to refine critical parameters, including the learning rates of the actor and critic networks, the gamma discount factor—which determines the reward time horizon—and the neural network architecture, defined by the number of layers and neurons per layer. These tests were made by applying genetic neural networks to 10% of the data set to determine optimal parameter settings.

The final model was selected based on its performance in time series cross-validation, its optimization of cumulative reward (maximizing profit or minimizing losses), and its stability across different time windows. The model architecture was tested with various network sizes and configurations, ultimately choosing the one that offered the best performance with the lowest reward volatility.

### Assessment metrics

In this article, the performance of the agent was assessed using metrics including average profit ( $AverageProfit$), average loss ( $AverageLoss$), and the stability of cumulative rewards across various time periods and data window configurations. We used the following evaluation metrics to measure the performance of the RL actor-critic model in reducing capital loss and managing risk in the gold, euro, oil, and Dow Jones markets:

The average profit, $AverageProfi{t_t}(n)$, refers to the average profit obtained by the agent in the $n$ episodes where it makes successful transactions. This is a fundamental metric in a financial context, as it indicates how much capital the agent generates on average when making the right decisions (buying at a low price and selling at a high price). The $AverageProfi{t_t}(n)$ is relevant because the agent’s main objective is to maximize profits in the long run while trading in volatile environments across different markets. RL seeks to optimize the agent’s policy to increase rewards over time. A high average profit suggests that the agent is learning to identify profitable buy/sell opportunities and is making consistent decisions to maximize the return on each transaction.

The average loss, $AverageLos{s_t}(n)$, measures the average losses incurred by the agent in $n$ episodes where it makes erroneous decisions (buying at high prices and selling at low prices). This is crucial for assessing whether the agent is successfully minimizing losses over time, which is one of the main objectives of this research. The $AverageLos{s_t}(n)$ is a key metric in financial risk management. In this environment, trading errors can lead to significant capital losses, so it is vital that the trader not only maximizes profits but also minimizes the impact of incorrect decisions. A low $AverageLos{s_t}(n)$ indicates that the trader is better at managing risks and avoiding trades that could result in large losses. Minimizing the average loss helps preserve capital and avoid large drawdowns, which is critical to the stability of an investment strategy.

### Experiments results

**Figure 10:**
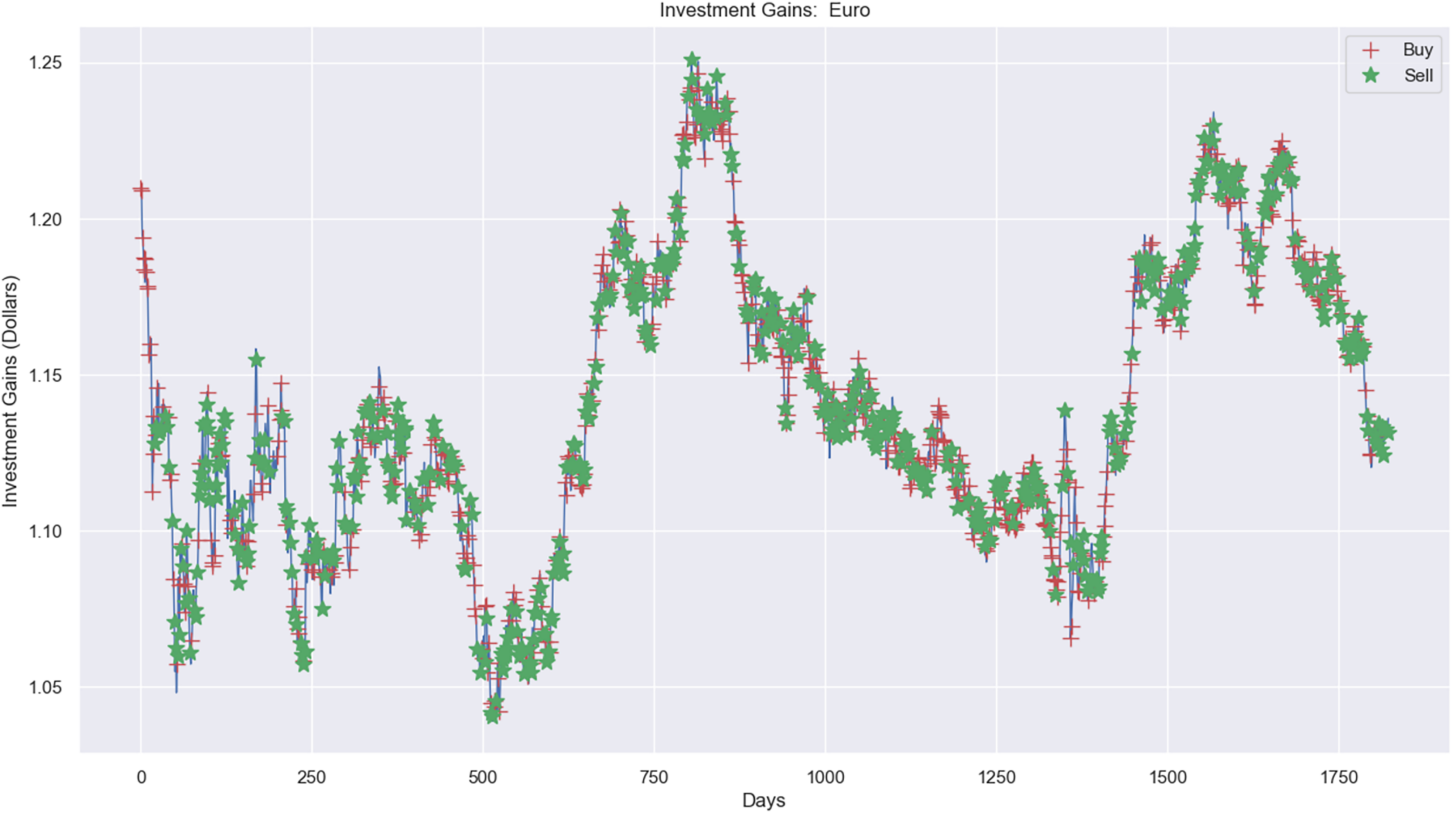
Investment actions (buy and sell) in Euro stocks (between 2015 and 2021).

**Figure 11:**
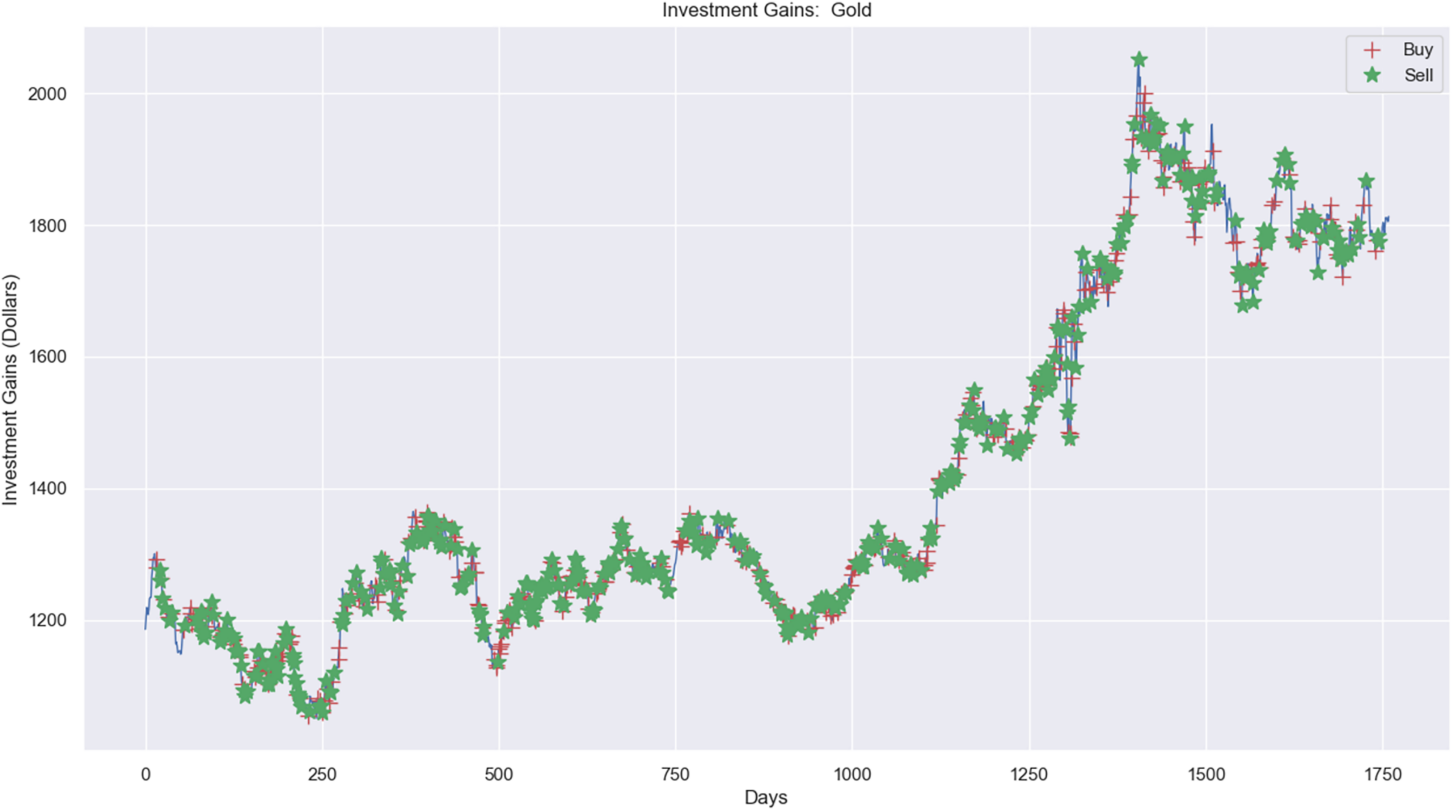
Investment actions (buy and sell) in gold stocks (between 2015 and 2021).

**Figure 12:**
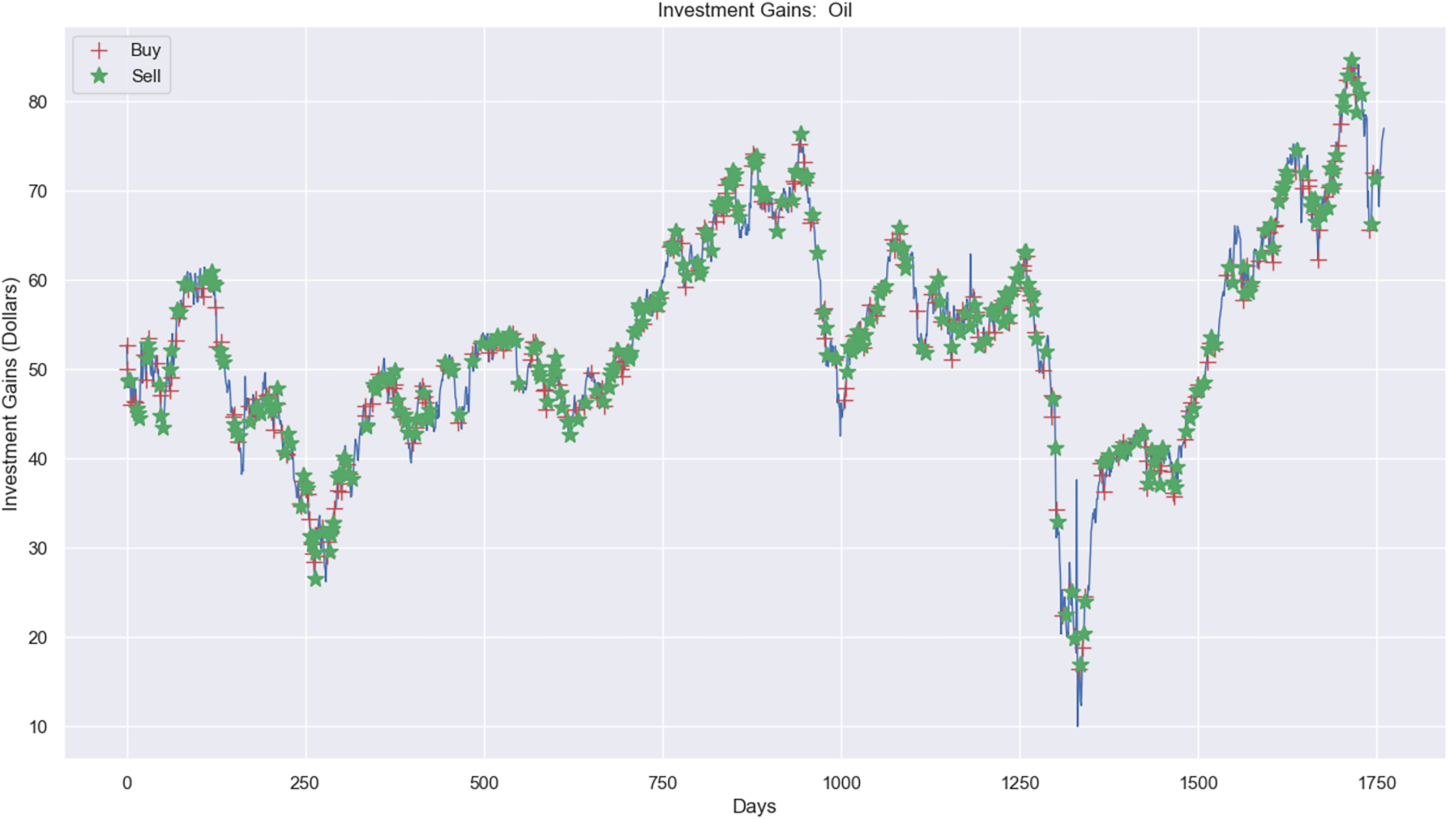
Investment actions (buy and sell) in crude oil stocks (between 2015 and 2021).

Experimental results, as shown in Figs. 10, 11, and 12A, present simulations conducted over the training (5 years) and testing (2 years) periods. These simulations utilize the RL model and 1-year window data for the Euro, gold, and crude oil stock markets. The figures depict investment actions in the market, including selling stocks (red cross), buying stocks (green star), and holding (no marker) stocks.

The case of the Euro market (Fig. 10) presents the investment simulation, where the model RL carried out 553 sell actions, 697 buy actions, and 573 hold actions. Figure 10 shows the investment price progress from $20,000 to $19,834 and a loss capital of $166.

Figure 11 presents the investment experiment in the gold market using RL. There were 437 sell actions, 437 buy actions, and 886 hold actions in the simulation. The profit from applying RL in the simulation amounted to $1,624.19, increasing from $20,000 to $21,624.19, as depicted.

In contrast, Fig. 12A shows the investment experiment in the crude oil market, where it carried out 313 sell actions, 320 buy actions, and 1,128 hold actions. The profit was $1.06, where the investment price process was from $20,000 to $20,001.06, applying the RL model.

A notable finding was the model’s ability to achieve higher profits in the crude oil market, particularly when utilizing semi-annual data windows. Despite the market’s high volatility, the profits exceeded expectations, highlighting the effectiveness of the adaptive data structure in managing rapid price fluctuations.

The results were obtained for each market over a test period of 2 years with annual, semiannual, and quarterly windows. To assess the proposed policies, the model computes the investment value, using the initial $Initia{l_{price}}$ and current $Curren{t_{price}}$ investment price in equitation 6, where the value is more than 0 $profit = value$ and if $value$ is less than 0 $loss = value$.

(6) $$value = {{Curren{t_{price}} - Initia{l_{price}}} \over {Initia{l_{price}}}}$$

The values of profit and loss obtained in this experiment are calculated with $AverageProfi{t_t}(n)$ and $AverageLos{s_t}(n)$. Average profit is the total profit divided by the number of transactions $n$ in $t$ time, as shown in Eq. (7). This value measures how much profit an investor makes on average per trade.

(7) $$AverageProfi{t_t}(n) = {{AverageProfi{t_{t - 1}}(n)(n - 1) + profi{t_t}} \over n}$$

On the other hand, the average loss is the total loss divided by the number of transactions $n$ in time $t$, as shown in Eq. (8).

(8) $$AverageLos{s_t}(n) = {{AverageProfi{t_{t - 1}}(n)(n - 1) + los{s_t}} \over n}$$

Tables 6 and 7 present the $AverageProfit$, obtained after applying the dynamic data window for each market. For crude oil, we obtained an annual average profit of 4.01%, smaller than the semester of 5.01% (4.78% and 5.24%) and the quadrimester one of 4.48% (4.18%, 4.59% and 4.66%). Similar results were obtained for the gold market, with higher profits in the semester of 4.61% (4.35% and 4.87%) and quarterly 4.24% (4.03%, 4.21% and 4.48%) windows than in the annual one of 4.11%. Finally, for the Euro market, we obtained an annual average profit of 2.37%, a semi-annual profit of 2.40% (2.39% and 2.41%), and a quarterly profit of 2.30% (2.22%, 2.17%, and 2.50%). Thus, in this experiment, the best data window seemed to be the semiannual one, which obtained better profit in every period in the stock market. Globally, the model generated significant profits in approximately 70.83% of tests and losses in only 29.16% of them.

**Table 6:** Experiment profit results for annual, semiannual, and quadrimester data windows.

Metrics	Periods	Crude oil	Gold	Euro
$AverageProfi{t_t}(n)$	Annual	4.01	4.11	2.37
	Semester 1	4.78	4.35	2.39
	Semester 2	5.24	4.87	2.41
	Quadrimester 1	4.18	4.03	2.22
	Quadrimester 2	4.59	4.21	2.17
	Quadrimester 3	4.66	4.48	2.50
$AverageLos{s_t}(n)$	Annual	0.01	0.02	0.03
	Semester 1	0.21	0.32	0.03
	Semester 2	0.22	0.42	0.05
	Quadrimester 1	0.18	0.36	0.02
	Quadrimester 2	0.16	0.41	0.01
	Quadrimester 3	0.21	0.33	0.03

**Table 7:** Average profit and loss results of simulations in the 2-year testing period.

	Periods	Crude oil	Gold	Euro
$AverageProfi{t_t}(n)$	Annual	4.01	4.11	2.37
	Semester	5.01	4.61	2.40
	Quadrimester	4.48	4.24	2.29
$AverageLos{s_t}(n)$	Annual	0.01	0.02	0.03
	Semester	0.21	0.37	0.04
	Quadrimester	0.18	0.37	0.02

The proposed RL model obtained an $AverageProfit$ of Euro 2.35%, gold 4.32%, and crude oil 4.50% in simulations tests. The obtained results had a Crude Oil profit between 4.01 and 5.01 in tests with an earned capital of $802 (annual), $1,002 (semester), and $896 (quarter). On the other hand, we have obtained the crude oil average loss between 0.01% and 0.21% with $2 (annual), $42 (semester), and $36 (quadrimester). The highest $AverageProfit$ values were obtained with the crude oil and gold market’s 6-month version, where the two first markets received profits, although the third market losses were primarily reduced by more than 1%, as shown in Table 7.

**Figure 13:**
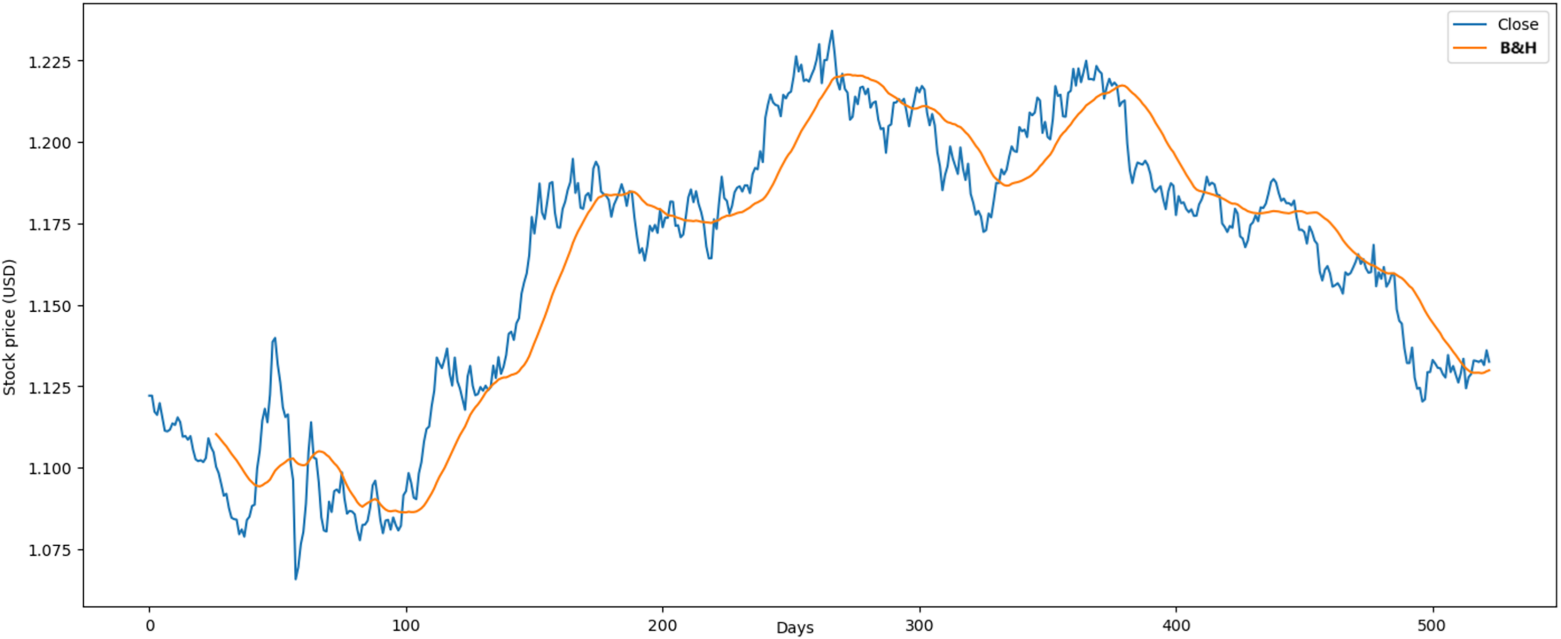
Application of B&H strategy in Euro market.

These results were favorable in the simulations compared with related studies and buy-and-hold strategy (B&H) (Atsalakis et al., 2019). In the Euro case, we compared with Chakraborty (2019), with the following results. First, the annualized loss of our RL model was 0.03% *vs* 9.81%, which decreased the $AverageLoss$ close to zero. In contrast, our model obtained is similar to Chakraborty (2019), with an average profit of 2.37 % *vs* 2.88% in the annual period. The results obtained through the B&H strategy showed an $AverageProfit$ of 0.007% and an $AverageLoss$ of 0.004% over a span of 2 years as shown in Fig. 13. These findings validate the optimal performance of our RL model.

The reduction in average loss achieved by the model reflects its capacity to minimize financial risks and enhance investment stability. For example, during the test phase, the model successfully reduced losses in the Euro market to 0.03%, demonstrating its effectiveness in managing risk. This metric underscores the model’s ability to mitigate suboptimal decisions and preserve capital, even in volatile market conditions.

**Figure 14:**
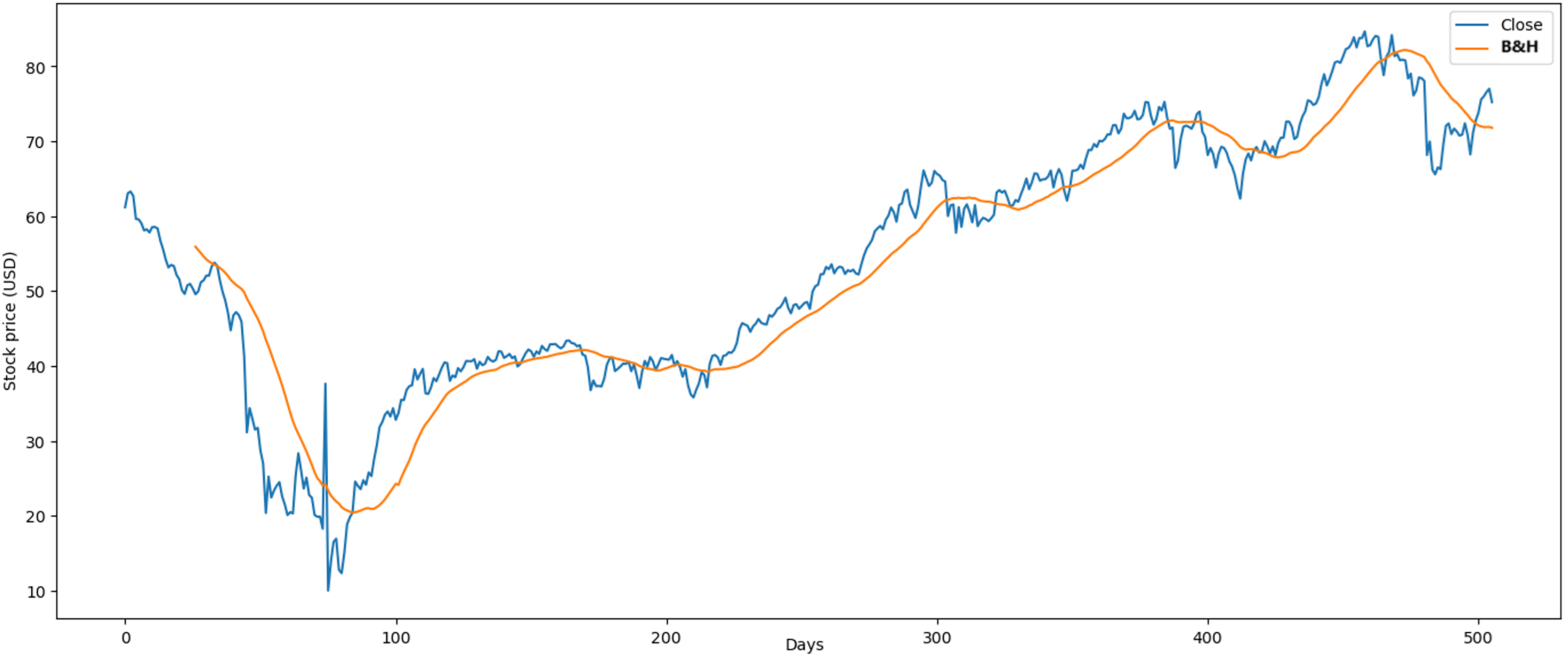
Application of B&H strategy in crude oil market.

The proposed RL model obtained optimal results in the crude oil case, where the average profit was 5.01% and Chakraborty (2019) study was 4.09%. The market behavior maintained a downward trend over long periods of time. In the market, many similar investment operations were made where it was easier to learn for the agent. The results obtained through the B&H strategy (Fig. 14) showed an average profit of 0.074% and an average loss of 0.068% where our RL model obtained higher results.

**Figure 15:**
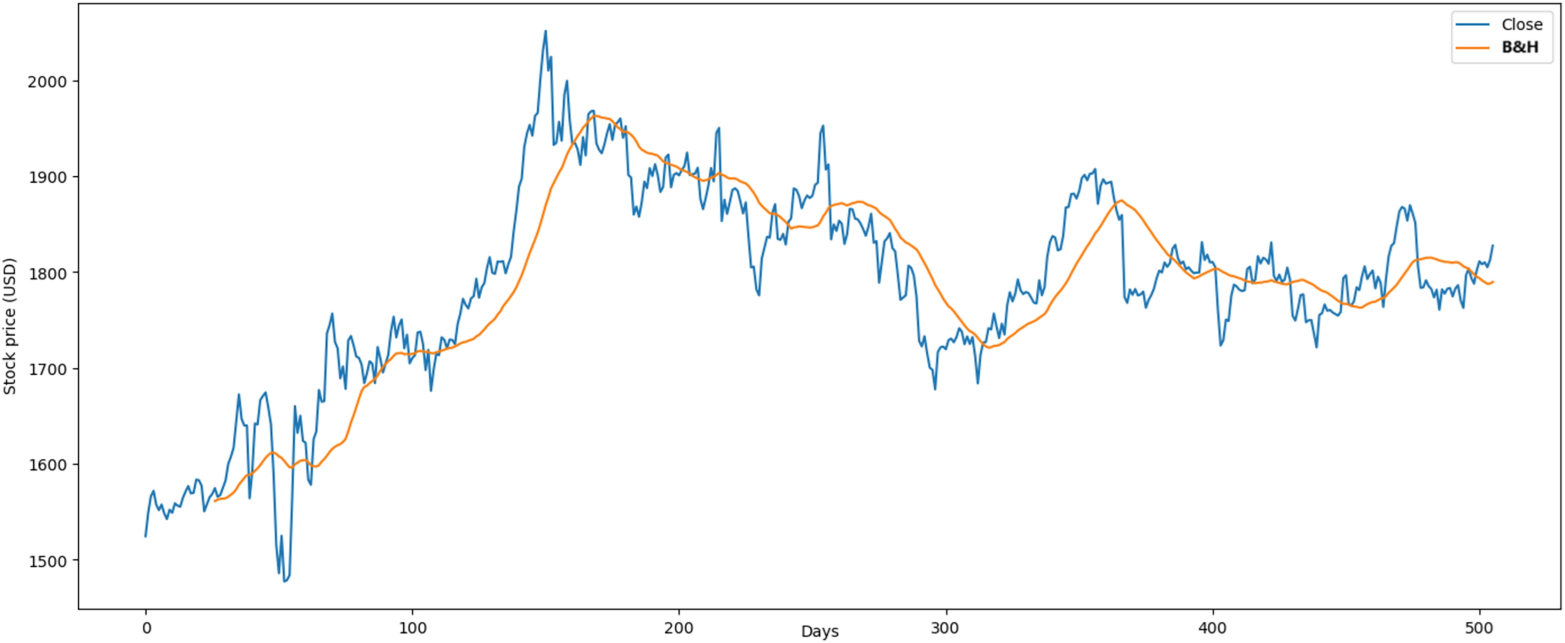
Application of B&H strategy in gold market.

In the Gold case, we compared the following results with Hirchoua, Ouhbi & Frikh (2021). The annualized average profit of our RL model was 4.11% *vs* 4.93%. Second, our model in the semester obtained 4.61%. Thus, our RL model had optimal profit in this period due to the adaptable data structure proposed more than Hirchoua, Ouhbi & Frikh (2021). Finally, the results obtained through the B&H strategy in the gold case were higher and showed an average profit of 0.064% and an average loss of 0.068% as shown in Fig. 15.

Transaction costs in the stock market depend on each broker in the U.S., where we have analyzed the fees of four brokers. These fees include stock commission ($0), Section 31 fees (ranging from $0.01 to $0.003 per $1,000), and Trading Activity fees (for selling, buying, and holding stocks, ranging from $0.03 to $0.05 per transaction). On the other hand, investment analysis costs range from $45 to $65 per transaction; however, this expense is not necessary in our proposal. Table 8 presents the fee commissions for each market during the tests of the RL model proposal, with crude oil at $70.88, gold at $88.60, and Euro at $141.76. These values represent less than 1% of the investment capital, making the involvement of an expensive investment analyst unnecessary.

**Table 8:** Section 31 (S31), trading activity (TA) and stock commission (SC) fee in the United States of America of crude oil, gold, and Euro market applying actor-critic RL model proposed.

	S31 fee	TA fee	SC fee	Total
Crude oil	$0.03	$0.01	$0	$70.88 (0.35%)
Gold	$0.03	$0.02	$0	$88.60 (0.44%)
Euro	$0.03	$0.05	$0	$141.76 (0.71%)

Distinct patterns were identified in the behavior of the three assets. The crude oil market exhibited pronounced volatility and frequent short-term price spikes, necessitating rapid adjustments by the agents. In contrast, the gold market demonstrated relative stability, characterized by gradual trends primarily driven by macroeconomic factors. The Euro market displayed intermediate volatility, reflecting the interplay between regional economic policies and global currency dynamics. These observations underscore the model’s ability to adapt effectively to diverse and dynamic market conditions. The model adapts to sudden market changes through the actor-critic framework, which facilitates real-time policy updates based on observed rewards. The integration of an adaptive data window structure ensures that agents are trained using both historical and recent data, enhancing their ability to respond effectively to abrupt shifts resulting from geopolitical events or economic shocks.

**Figure 16:**
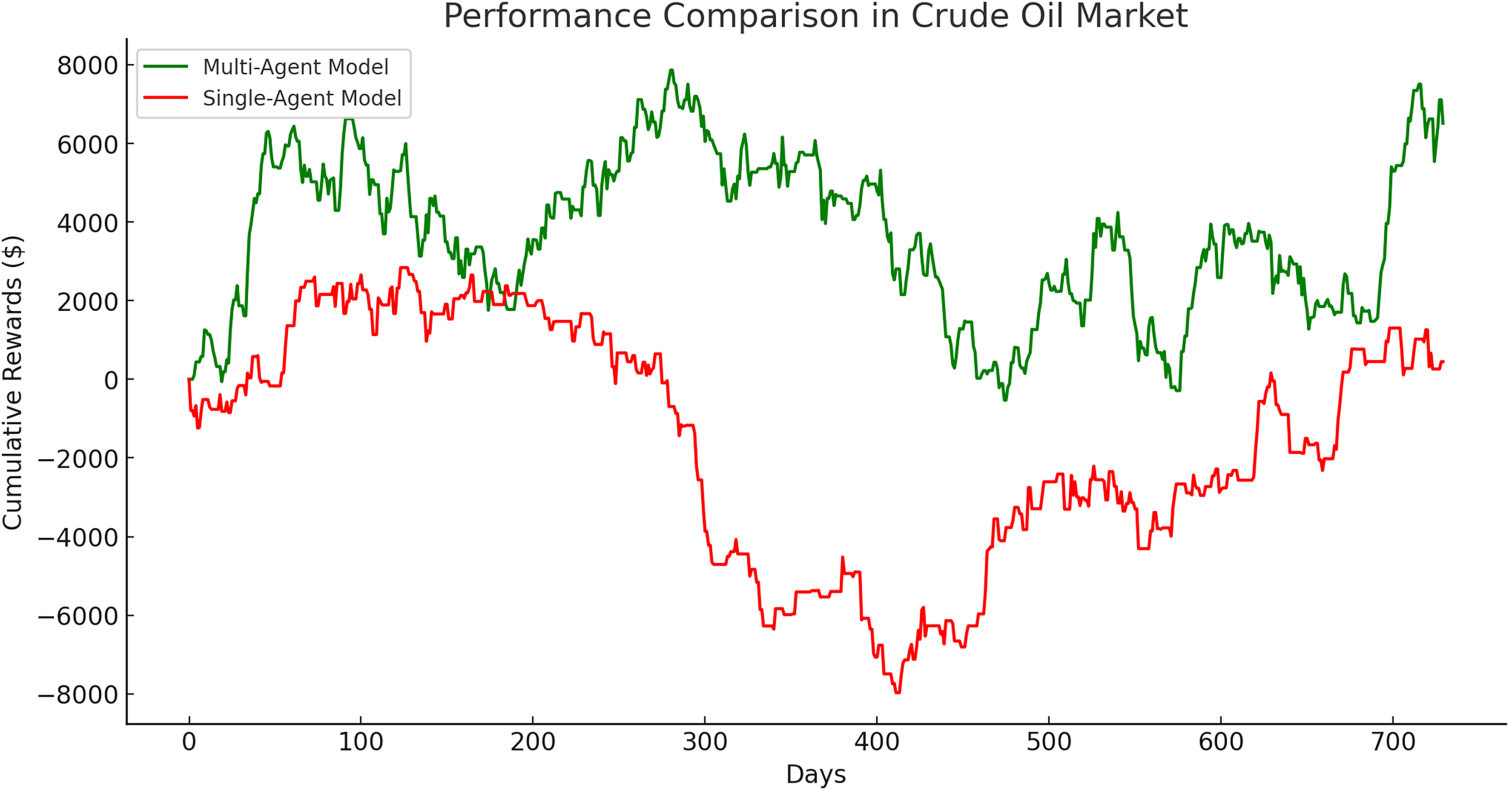
Performance comparison in crude oil market between multi-agent and single-agent.

The single-agent model exhibited its poorest performance in the crude oil market, with a significantly lower cumulative gain of $449.57 compared to its performance in the gold and Euro markets. This disparity is evident in Fig. 16, which illustrates a clear divergence in cumulative rewards between the multi-agent and single-agent models in this market.

To validate the effectiveness of the proposed multi-agent model, a comparative analysis was conducted against a single-agent model across three financial markets: Gold, crude oil, and Euro. The results, presented in Table 9, consistently demonstrate the superior performance of the multi-agent approach in terms of final cumulative rewards, absolute differences, and percentage improvements.

**Table 9:** Comparison of multi-agent (M-A) and single-agent (S-A) model performance across markets.

Market	M-A final reward ($)	S-A final reward ($)	Difference ($)	Improvement (%)
Gold	12,273.12	3,646.93	8,626.19	236.53
Crude oil	6,512.58	449.57	6,063.02	1,348.64
Euro	9,402.83	2,496.58	6,906.25	276.63

The multi-agent model outperformed the single-agent model across all markets. In the gold market, it achieved a 236.53% improvement, underscoring its capability to make efficient decisions in less volatile conditions. Similarly, in the Euro market, the multi-agent approach demonstrated a 276.63% improvement, further highlighting its adaptability and precision in moderately volatile environments.

The crude oil market presented the largest performance gap, with the multi-agent model outperforming the single-agent model by an extraordinary 1,348.64%. The single-agent model achieved only a modest cumulative reward of $449.57, reflecting the challenges posed by the high volatility of this market. In contrast, the multi-agent model demonstrated remarkable adaptability and robustness, achieving a cumulative reward of $6,512.58. These results emphasize the multi-agent system’s ability to manage rapid price fluctuations and navigate complex market dynamics effectively.

The superior performance of the multi-agent model can be attributed to its modular design, which leverages specialized agents trained on specific time windows. This approach enables the model to capture market-specific patterns, reduce training time, and enhance decision-making precision. Consequently, the multi-agent system excels at risk management and mitigates the impact of suboptimal actions, as demonstrated by the substantial performance differences across all markets.

These findings provide compelling empirical evidence supporting the claim that the multi-agent model represents a more effective approach to financial trading, particularly in volatile market conditions. Its dynamic adaptability and ability to optimize investment strategies make it a valuable tool for modern financial systems.

The actor-critic model’s adaptive data windowing enables effective navigation of volatile financial markets, such as crude oil, characterized by rapid price fluctuations due to geopolitical and economic factors. This adaptability allows the model to dynamically adjust its training data windows, identifying short-term patterns crucial in such environments. Consequently, the actor-critic model achieves superior average gains, notably 5.0% in crude oil, while minimizing losses more effectively than traditional methods like DQN, which consistently underperform across various markets (Table 10).

**Table 10:** Performance comparison of reinforcement learning methods across markets.

Metric	Market	Actor-Critic	PPO	DQN
Average gains (%)	Euro	2.4	2.1	1.8
	Gold	4.6	4.2	3.8
	Crude oil	5.0	4.8	4.2
Average losses (%)	Euro	0.03	0.05	0.07
	Gold	0.37	0.42	0.48
	Crude oil	0.21	0.25	0.31
Convergence time (episodes)	Euro	1,200	1,500	2,000
	Gold	1,000	1,300	1,800
	Crude oil	950	1,200	1,700
Stability (Standard deviation)	Euro	0.8	1.2	1.5
	Gold	0.6	1.0	1.4
	Crude oil	0.5	0.9	1.2

Quantitatively, as Table 10 shows, the actor-critic model demonstrates faster convergence, requiring only 950 to 1,200 episodes across all markets, compared to DQN’s extended convergence time of up to 2,000 episodes. Additionally, it exhibits enhanced stability, indicated by a lower standard deviation in rewards per episode, underscoring its reliability in volatile scenarios where traditional models often falter.

These findings highlight the actor-critic model’s proficiency in adjusting to dynamic market conditions through adaptive data windowing. This leads to improved financial performance and computational efficiency. The model’s robustness and stability make it a valuable tool for automated trading in complex and rapidly changing financial markets.

### Limitations/validity

Limitations of this approach, such as reliance on historical data, risks of overfitting, and simplifications in operating costs, are factors that could influence the model’s performance in real market conditions. However, validation methods such as time-splitting and comparisons with traditional strategies provide credibility to the results and demonstrate that the model can be effective in reducing losses in market scenarios with moderately predictable behaviors.

The proposed model offers significant practical applications for financial institutions and individual investors. By minimizing losses and optimizing profits in volatile markets, it can be effectively integrated into portfolio management strategies, serving as an automated tool for real-time investment decision-making. Furthermore, its adaptive capabilities enable it to respond to market fluctuations, making it particularly well-suited for dynamic and unpredictable global financial environments. The model demonstrates robust performance in both bull and bear markets, owing to its dynamic adaptability to changing market conditions. By leveraging short-term patterns through the adaptive data window structure, the model maintains consistent performance, effectively reducing losses during downward trends and capitalizing on upward momentum in bull markets.

## Conclusions and future work

Stock market trading deals with dynamic environments affected by speculation and other external factors, including the impact of sociopolitical changes on the markets. A significant problem in efficiently investing in a specific market is knowing more profoundly the market behavior in a particular season (period) when the prices of stocks go up or down according to demand. Frequently, banks and companies create and train a unique investment model with all historical information on markets, which may lead to investment actions with higher error rates and fewer gains. Our model proposes a dynamic data window structure to train each investment agent to improve the $AverageProfit$. The data window could change over time and adapt to a specific period, better detecting the market behavior. A key distinguishing feature of our model is the integration of an adaptive data window structure, which optimizes learning for specific time periods, in contrast to traditional approaches that rely on fixed data windows. This methodology enables investment agents to adapt their learning processes to the temporal dynamics of the market, thereby enhancing both accuracy and learning efficiency. This adaptability is particularly advantageous in dynamic financial environments, where market patterns can shift rapidly.

Our approach demonstrates significant advantages over established RL methods, such as deep Q-networks (DQN) and proximal policy optimization (PPO). For instance, while DQN models are designed for discrete action spaces and often face challenges in continuous market environments, our actor-critic framework is well-suited for handling dynamic decision-making. Additionally, unlike PPO, which relies on conservative strategies to explore new policies, our model employs an advantage-based policy that facilitates more efficient learning by effectively balancing exploration and exploitation.

The multi-agent approach assigns individual agents to specific data windows, enabling specialized and more efficient learning. In contrast, a single agent trained across all time periods risks overgeneralization, potentially reducing accuracy in capturing specific market dynamics. By allowing each agent to develop optimal policies tailored to its assigned time range, this design enhances the overall robustness and adaptability of the model.

The results showed that the proposed RL seems an exciting option for implementation in the stock market due to its profit rate and efficient adaptation to a changing environment. An essential characteristic of our proposal was defining the optimal data window size for each market, which was executed in the experimental phase with moderate profits. This data window, combined with RL, facilitates better results and improves investment performance with low risks. This combination allows knowing the best configuration of the neural networks for the training of the RL model proposed, the number of agents for implementation, and the optimal size of the data windows for each market (environment). In addition, the technological resources used are lower due to the reduced data for each agent learning and in all experiment phases.

Political and economic factors significantly influence the performance of the model by introducing variability in market trends and volatility. Geopolitical conflicts, regulatory changes, and economic crises can profoundly affect price behavior, necessitating dynamic adaptation by the model. Through the use of an adaptive data window structure and the actor-critic reinforcement learning approach, the model effectively mitigates risks associated with abrupt market shifts, thereby enhancing decision-making in unpredictable and volatile conditions.

Automated trading systems inherently carry risks, including biases in historical data and unforeseen outcomes in low-liquidity markets. The proposed model addresses some of these challenges by minimizing exposure to erroneous decisions through rigorous cross-validation and comprehensive robustness evaluations. From an ethical perspective, we emphasize the importance of responsible implementation, ensuring adherence to financial regulations and explicitly discouraging the use of algorithms for market manipulation.

In future work, we intend to use other data related to the stock market, industry, and social networks to identify future market changes. We also intend to apply the game theory and Bayesian models to optimize decision results. Future research directions may involve the integration of advanced macroeconomic indicators and the adoption of hybrid methodologies, such as game theory, to further enhance decision-making processes in highly competitive and dynamic environments. Additionally, the RL model could be expanded to incorporate complex market indicators by extending its input feature space to include variables such as economic indices, geopolitical event data, and sentiment analysis derived from news and social media. The inclusion of these features is expected to augment the model’s ability to detect nuanced patterns, thereby improving its accuracy and robustness in decision-making. The model can be extended to encompass additional asset classes, such as cryptocurrencies and tokenized real estate, to evaluate its applicability in alternative markets.

One of the most challenging aspects of this research was the design of the adaptive data window structure, which necessitated extensive experimentation to determine optimal configurations under varying market conditions. Additionally, achieving a balance between computational efficiency and model accuracy presented a significant challenge.

Future research efforts should prioritize understanding the unique dynamics of financial markets while exploring advanced machine learning methodologies, such as reinforcement learning. Emphasis should also be placed on developing robust validation frameworks and addressing ethical considerations to ensure the creation of reliable and impactful financial models.

## Supplemental Information

10.7717/peerj-cs.2690/supp-1 Supplemental Information 1 Datasets and Matlab code.

## Additional Information and Declarations

### Data Availability

The following information was supplied regarding data availability:

The raw data and computer code are available in the Supplemental Files.
